# The placental battlefield: viral strategies and immune countermeasures

**DOI:** 10.3389/fimmu.2025.1667601

**Published:** 2025-12-17

**Authors:** Ruby Dhar, Sunil Singh, Om Saswat Sahoo, Nilesh Chandra, Anamta Gul, Indrani Mukherjee, Shreyashi Karmakar, Mohammed Amanullah, Subhradip Karmakar

**Affiliations:** 1 Department of Biochemistry, All India Institute of Medical Sciences New Delhi, New Delhi, India; 2 Department of Biotechnology, National Institute of Technology Durgapur, West Bengal, Durgapur, India; 3 Division of Discovery Research, Indian Council of Medical Research, New Delhi, India; 4 Department of Pediatrics, Division of Infectious Diseases and Immunology, University of Miami, Miami, FL, United States; 5 Department of Obstetrics and Gynaecology, Employees State Insurance-Post Graduate Institute of Medical Sciences and Research (ESI-PGIMSR), Employees State Insurance Corporation (ESIC) Medical College, Joka, West Bengal, India; 6 Department of Clinical Biochemistry, College of Medicine, King Khalid University, Abha, Saudi Arabia

**Keywords:** placenta, viral infection, maternal-fetal transmission, pregnancy complications, antiviral therapy, fetal development

## Abstract

The placenta plays an essential role in connecting the maternal and fetal environments. It acts as both a protective barrier and a selective transport system during pregnancy. Despite its importance, we still do not fully understand how the placenta responds to viral infections, leaving a notable gap in maternal-fetal medicine. This review looks at how viral pathogens interact with placental tissue. It explores how viruses are transmitted, how the placenta’s immune system responds, and how infections affect pregnancy outcomes. We examined recent findings on how viruses can penetrate placental barriers, the molecular processes that lead to placental damage, and the long-term effects on fetal development. We gathered evidence from SARS-CoV-2, Zika virus, cytomegalovirus, and other viral infections to highlight common pathways and point out possible treatment targets. As new viral threats continue to challenge healthcare systems worldwide, understanding placental virology is crucial for safeguarding both maternal and fetal health. This review outlines potential future research paths and emphasizes the urgent need for placenta-specific antiviral strategies as new infectious diseases emerge.

## 1 Introduction

The human placenta is one of nature’s most complex biological interfaces. It manages the exchange of nutrients, gases, and waste between the mother and fetus while also protecting the fetus from harmful pathogens (1, 2). This remarkable organ is often referred to as a “black box” because studying its function during human pregnancy is challenging. It has developed complex mechanisms that balance the need for essential transport with protection against infections (3). However, the rise of new viral pathogens and the return of known ones have exposed significant gaps in our knowledge of placental virology and its effects on pregnancy outcomes.

Viral infections during pregnancy have long been recognized for their potential to cause serious fetal and neonatal complications, as evidenced by congenital infections such as rubella, cytomegalovirus, HIV, and Zika virus (4–7). These pathogens can disrupt fetal development, leading to long-term consequences for brain development, cardiovascular health, and immune system maturation. The COVID-19 pandemic, however, drew renewed global attention to the possibility of mother-to-fetus viral transmission, highlighting our limited understanding of how the placenta responds to diverse viral challenges (8). The placenta has always been known as a barrier against many pathogens. However, growing evidence shows that many viruses have found ways to get past these placental defenses. This can result in congenital infections, complications during pregnancy, and negative outcomes for the fetus. The effects of viral infection in the placenta go beyond immediate issues during pregnancy. They can also affect long-term brain development, heart health, and how the immune system develops in children (9).

Recent technological advances in placental research, including single-cell sequencing, imaging techniques, and *in vitro* models, have started to shed light on the molecular mechanisms behind viral-placental interactions. These tools reveal the diverse cellular landscape of the placenta, the changing nature of placental immune responses, and the different strategies viruses use to establish infection and avoid host defenses (10). Understanding these mechanisms is essential for creating targeted interventions to protect both maternal and fetal health during viral epidemics and pandemics.

This comprehensive review aims to synthesize current knowledge regarding viral infections of the placenta, examining the mechanisms of viral transmission, placental pathology, and pregnancy outcomes across different viral pathogens. We explore the unique immunological environment of the placenta, analyze virus-specific pathogenic mechanisms, and discuss the clinical implications of placental viral infections. Furthermore, we identify key knowledge gaps and propose future research directions that may lead to improved prevention and treatment strategies for viral infections during pregnancy.

## 2 Placental structure and function: the foundation for understanding viral susceptibility

### 2.1 Architectural organization and cellular composition

The human placenta exhibits a complex architectural organization that directly influences its susceptibility to viral infections and the patterns of viral transmission to the fetus. The placental barrier consists of multiple cellular layers that separate maternal and fetal blood, including the syncytiotrophoblast, cytotrophoblast, villous stroma, and fetal capillary endothelium (11). The maternal–fetal interface is further structured by the decidua basalis, which contains decidual stromal cells, NK cells, and macrophages, forming a cellular and stromal architecture that regulates maternal immune tolerance while also providing potential entry portals for pathogens (12, 13). Each of these layers presents unique vulnerabilities and resistance mechanisms to viral pathogens. The syncytiotrophoblast, a multinucleated layer formed by the fusion of cytotrophoblasts, represents the primary interface with maternal blood and serves as the first line of defense against viral invasion (14). This specialized epithelium expresses numerous viral receptors and co-receptors, making it a critical determinant of viral tropism and transmission efficiency. Underlying cytotrophoblasts serve as proliferative progenitors and potential viral reservoirs, whereas extravillous trophoblasts (EVTs) migrate into the decidua and along spiral arteries, remodeling maternal vasculature and creating additional sites where maternal pathogens can access fetal tissues (15). The syncytiotrophoblast’s continuous renewal through cytotrophoblast fusion may serve as a protective mechanism against persistent viral infections. Still, this same process can facilitate viral spread if the underlying cytotrophoblast pool becomes infected (9, 14). The maternal vasculature in virtue of the spiral artery remodeling converts high-resistance vessels into low-resistance, high-flow channels, not only ensuring maternal perfusion of the intervillous space but also creating conduits whereby maternal blood-borne pathogens can reach the villous surface and potentially infect trophoblasts (15, 16). Spiral artery remodeling is a tightly regulated process driven primarily by EVTs. Endovascular EVTs invade and replace the endothelial lining of spiral arteries, while interstitial EVTs degrade surrounding smooth muscle and extracellular matrix components, enlarging vessel lumens and decreasing vascular resistance. Decidual immune cells, particularly uterine NK cells and macrophages, secrete cytokines and growth factors that guide EVT invasion and support vascular adaptation. While these changes are essential for adequate maternal blood flow to the placenta, they also create anatomical conduits that maternal pathogens can exploit. Viruses such as CMV and ZIKV have been shown to infect EVTs and use remodeled spiral arteries to approach the intervillous space, highlighting a dual role for spiral artery remodeling in supporting fetal nutrition while inadvertently facilitating pathogen delivery to fetal compartments (17, 18).

The syncytiotrophoblast forms the outermost layer of placental villi and is directly exposed to maternal blood, making it the first line of defense against viral pathogens. This multinucleated epithelium is generated through continuous fusion of underlying cytotrophoblasts, a process mediated by fusogenic proteins such as syncytin-1 and syncytin-2, as well as the GCM1 transcriptional network (11). The dynamic turnover of syncytiotrophoblasts through cytotrophoblast fusion and syncytial shedding serves both to maintain barrier integrity and to limit accumulation of intracellular pathogens. Metabolically, syncytiotrophoblasts exhibit high mitochondrial activity and glycolytic flux, enabling sustained ATP production to support active transport, hormone biosynthesis, and barrier maintenance (19). They secrete pregnancy-critical hormones, including human chorionic gonadotropin (hCG), placental lactogen, and progesterone, which not only regulate maternal physiology but also modulate local immune tolerance. The dense microvillous surface and lipid-raft–rich membranes of syncytiotrophoblasts, together with active shedding of syncytial knots, create both structural and immunological niches that can be exploited by pathogens. This includes potential docking or entry sites for viruses, as well as systemic dissemination of apoptotic fragments into maternal blood (20). The dense microvillous surface of syncytiotrophoblasts maximizes exchange of gases, nutrients, and metabolites, while simultaneously serving as the interface where viral attachment and entry occur. Structurally, syncytiotrophoblasts undergo constant renewal and remodeling. Microvillous membranes contain abundant lipid rafts and glycoproteins that act as potential viral docking sites. Viral exploitation of these domains has been demonstrated for Zika virus (via AXL and TYRO3 receptors), HIV-1 (via galactosylceramide and CCR5/CXCR4), SARS-CoV-2 (via ACE2 and TMPRSS2), and cytomegalovirus (via integrins and PDGFRα) (8, 21–23). Moreover, syncytial knots, aggregates of pyknotic nuclei formed during apoptosis, represent natural sites of structural weakening, which may facilitate viral penetration or enhance susceptibility under inflammatory conditions (24, 25). Shedding of apoptotic syncytial fragments into the maternal circulation provides an additional route for systemic immune activation and potentially disseminates virally infected material (26). Thus, the syncytiotrophoblast is not a static barrier but a metabolically active, dynamically renewed epithelium whose biogenesis, metabolic demands, endocrine functions, and structural remodeling collectively determine its susceptibility to and exploitation by viral pathogens.

Beneath the syncytiotrophoblast lies the cytotrophoblast layer, consisting of proliferative stem cells that maintain the syncytial layer and contribute to placental growth throughout pregnancy. These cells express different receptor profiles compared to the syncytiotrophoblast and may serve as viral reservoirs or sites of persistent infection. The villous stroma contains diverse cell populations, including mesenchymal cells, macrophages (Hofbauer cells), and fibroblasts, each with distinct susceptibilities to viral infection and roles in immune responses (27) (explained later). In addition, the coordinated presence of EVTs, stromal cells, and Hofbauer cells determines how efficiently maternal viruses can be trafficked from decidua to villi, emphasizing that placental susceptibility is both cell-type and spatially dependent (28).

### 2.2 Decidual architecture and viral entry portals

The decidua, representing the maternal compartment of the placenta, harbors abundant immune and stromal cell populations, including decidual macrophages, dendritic cells, NK cells, and T lymphocytes. Many viruses exploit these cells as Trojan horse carriers, facilitating translocation across the maternal–fetal interface. For instance, HIV-1–infected decidual macrophages can transmit virus to trophoblasts, while CMV establishes latency within decidual stromal and immune cells, providing a persistent reservoir (27). Zika virus (ZIKV) has also been detected in decidual macrophages and dendritic cells, highlighting the decidua as a primary site of viral entry and persistence (29). Structurally, decidua basalis contains spiral arteries, stromal fibroblasts, vascular smooth muscle cells, and a dense immune infiltrate, all of which create both immunological and anatomical gateways for infection (30, 31). Decidual NK cells, though abundant and critical for vascular remodeling, produce cytokines that viruses can co-opt to facilitate spread (30, 32). Decidual stromal cells act as long-lived niches for CMV and may interact with infiltrating maternal leukocytes to promote viral persistence (30, 32). These features collectively render the decidua not merely a passive uterine lining but an active interface where viral reservoirs form and viral dissemination into trophoblasts is initiated. EVTs invade the decidua and myometrium, directly interacting with maternal immune cells and extracellular matrix components. By expressing HLA-G and other immunomodulatory molecules, EVTs facilitate immune tolerance but simultaneously become targets for viral exploitation. EVTs are permissive to CMV and ZIKV, both of which can replicate within these cells and exploit their invasive routes to penetrate deeper into maternal tissues and gain access to fetal compartments (33). Spiral artery remodeling, mediated by EVTs, transforms high-resistance maternal vessels into low-resistance conduits, enhancing perfusion of the intervillous space. This vascular remodeling increases direct exposure of the syncytiotrophoblast to maternal blood and circulating viruses (34). CMV DNA has been detected in remodeled spiral arteries, and maternal viremia (HIV-1, parvovirus B19, ZIKV) correlates with higher risks of placental infection, underscoring spiral arteries as critical portals for viral delivery (27).

### 2.3 Additional cellular carriers and viral reservoirs

Infected maternal monocytes, lymphocytes, and dendritic cells traversing the decidua and intervillous space act as viral reservoirs and vehicles of vertical transmission. HIV-1–infected maternal monocytes have been demonstrated to cross the placental barrier and transmit virus to fetal macrophages. Hofbauer cells, resident macrophages of the villous stroma, are highly permissive to HIV-1, CMV, and ZIKV, supporting replication and amplifying local infection (27). Moreover, mesenchymal stromal cells and fibroblasts within the stroma may serve as secondary reservoirs that prolong viral persistence within the placental tissue. Despite its formidable defenses, the placental barrier exhibits anatomical weak points that viruses exploit. The syncytiotrophoblast expresses multiple viral receptors (e.g., AXL, DC-SIGN, ACE2), rendering it permissive to ZIKV, HIV-1, and SARS-CoV-2 (35). Microdisruptions in syncytial integrity, such as syncytial knots or apoptotic shedding, create portals for viral entry (26, 36). The dynamic cytotrophoblast pool, essential for syncytial renewal, may serve as a latent reservoir, as shown for CMV infection. Remodeling-associated vascular breaches and immune cell trafficking further provide direct conduits for viral dissemination. Collectively, these structural vulnerabilities viz., decidual immune reservoirs, invasive EVT pathways, remodeled spiral arteries, and stromal reservoirs, constitute an integrated network of entry points that viruses can co-opt to breach the maternal–fetal interface.

The placental vascular system creates unique conditions that influence the dynamics of viral transmission. Maternal blood flows through the intervillous space at relatively low velocities, resulting in prolonged contact times between viral particles and placental surfaces. This hemodynamic environment may facilitate viral attachment and entry while also enabling the accumulation of inflammatory mediators that can compromise placental function (37, 38). This low-shear environment favors viral attachment, receptor engagement, and potential transcytosis, while also enabling the local accumulation of cytokines and inflammatory mediators that may compromise barrier integrity and facilitate infection. The fetal vascular system within placental villi is separated from maternal blood by the placental barrier layers described above. However, breaks in barrier integrity due to viral infection, inflammation, or mechanical disruption can lead to direct maternal-fetal blood mixing, thereby enhancing the transmission risk. Understanding these vascular dynamics is crucial for predicting transmission probabilities and developing intervention strategies. Hemodynamic factors directly impact HIV-1 vertical transmission risk. Monocytes and dendritic cells carrying HIV-1 migrate across vascular compartments, acting as Trojan horses that deposit virus in the intervillous space (39). The low-flow intervillous milieu prolongs exposure of syncytiotrophoblast receptors to HIV-1 virions. Although vertical transmission of SARS-CoV-2 is relatively rare, vascular architecture provides plausible routes for fetal exposure. SARS-CoV-2 has been detected in maternal blood and, in some cases, within intervillous thrombi and maternal vascular malperfusion lesions (40). The virus binds to ACE2 and TMPRSS2, which are expressed on syncytiotrophoblasts and endothelial cells of the chorionic villi, particularly under hypoxic or inflammatory conditions. The low-shear intervillous environment may allow viral particles or extracellular vesicle–associated virions to accumulate and adhere to trophoblast surfaces (40). Additionally, SARS-CoV-2–infected maternal immune cells can traverse decidual vessels and infiltrate placental tissue, linking vascular trafficking with localized viral delivery. The hemodynamic characteristics of placental circulation critically determine parvovirus B19 pathogenesis. Unlike HIV-1 and SARS-CoV-2, B19 has a strong tropism for erythroid progenitor cells (41). Breaks in the placental barrier or maternal–fetal microtransfusions allow direct entry of maternal blood into fetal circulation, where B19 targets erythroid precursors in villous capillaries via the P antigen (globoside) receptor. This mechanism explains the association between maternal B19 viremia, placental vascular injury, and fetal hydrops (41). Collectively, these hemodynamic and cellular carriage pathways underscore the importance of placental vascular architecture as both a protective filter and a conduit for viral dissemination.

HIV exhibits tropism for erythroid precursors and mature erythrocytes through specific receptor-mediated interactions, including binding to complement receptor 1 (CR1/CD35) and Duffy antigen receptor for chemokines (DARC) (42, 43). This erythrocytic association facilitates viral persistence within the circulation and enables tissue-specific delivery through the microvascular network. The extended lifespan of erythrocytes (approximately 120 days) provides HIV with a stable reservoir for sustained viral dissemination, particularly relevant for transplacental transmission during pregnancy (42). The incorporation of viral particles within erythrocytic cellular compartments or surface association allows evasion of circulating neutralizing antibodies while maintaining viral viability during transit. This mechanism is particularly significant for understanding persistent viral infections and their capacity to establish tissue-specific reservoirs through selective vascular distribution patterns.

## 3 Mechanisms of viral transmission across the placental barrier

### 3.1 Receptor-mediated viral entry and viral receptors in the placenta

The placenta serves as a critical barrier between maternal and fetal circulation, regulating the transfer of nutrients, gases, and waste products while protecting the developing fetus from potentially harmful pathogens. However, this protective barrier is not impermeable to all infectious agents. Viral receptors expressed on placental cells represent key determinants of viral tropism and transmission across the maternal-fetal interface. While cellular receptor expression is a necessary prerequisite for viral entry, the presence of receptors alone does not guarantee productive viral infection (44). Productive infection requires successful completion of the entire viral lifecycle, including entry, uncoating, replication, assembly, and release, which depends on multiple host cell factors beyond receptor availability (45). These factors include cellular metabolism, transcriptional machinery, post-translational modification systems, innate immune responses, and cell cycle status (45, 46).

Viral entry into placental cells depends primarily on the expression of specific viral receptors and co-receptors on target cell surfaces. Different placental cell types exhibit distinct receptor profiles, resulting in tissue-specific patterns of viral susceptibility that influence transmission dynamics and pathogenesis. (**Table 1**) The syncytiotrophoblast has many viral receptors, such as ACE2 for SARS-CoV-2, AXL for the Zika virus, and various integrins that act as entry points for different viral families (8, 27). The way these receptors changes over the course of pregnancy can affect a person’s vulnerability to viral infections at different stages. Knowing these expression patterns is essential for predicting the risk of transmission and creating targeted treatments. Cytotrophoblasts and other placental cell types express different receptor repertoires, creating opportunities for viral persistence and spread within placental tissues even when the syncytiotrophoblast remains resistant (27). This heterogeneity in receptor expression contributes to the complex patterns of viral infection observed in placental tissues, highlighting the importance of studying individual cell populations rather than whole tissue samples.

**Table 1:** Placental cell types and their respective viral receptors.

Cell type	Primary receptors	Virus	Expression level	Functional/clinical relevance	Ref
Syncytiotrophoblast	ACE2 + TMPRSS2	SARS-CoV-2	High	Major site of viral entry from maternal blood; high ACE2/TMPRSS2 expression in late pregnancy correlates with increased placental infection and potential vertical transmission; implications for antiviral therapy targeting ACE2/TMPRSS2.	(69)
	AXL + Tyro3	ZIKV, DENV	Moderate-High	Facilitates flavivirus entry; linked to congenital Zika syndrome and fetal neurodevelopmental defects; guides antiviral development and placental-targeted vaccines.	(17, 70)
	α2β1, αvβ3 integrins	CMV	High	Critical for CMV attachment and internalization; contributes to congenital CMV infection and placental inflammation; informs passive immunization strategies to prevent congenital CMV.	(71)
Cytotrophoblast	ACE2 + TMPRSS2	SARS-CoV-2	Moderate	Serves as a reservoir for infection; contributes to restricted viral replication and may mediate limited maternal-fetal transmission; potential target for prophylactic antiviral intervention.	(72–74)
	Nectin-1	HSV-1/2	Moderate	Enables herpesvirus entry; potential source of congenital HSV infection if maternal viremia occurs; informs maternal antiviral therapy to reduce vertical transmission.	(75, 76)
Hofbauer Cells	DC-SIGN (CD209)	HIV	High	Promotes HIV capture and transmission within placental villi; implicated in vertical transmission risk; therapeutic target for reducing fetal exposure.	(77)
	CD163	HIV	High	Supports viral replication and immune evasion; contributes to fetal exposure to HIV; potential target for macrophage-directed antivirals.	(78)
Endothelial Cells	ACE2 + TMPRSS2	SARS-CoV-2	Variable	May facilitate viral trafficking into fetal circulation under inflammatory conditions; implicated in placental vascular pathology; relevant for maternal antiviral strategies and vascular protection.	(26)
	ICAM-1 (CD54)	Rhinovirus	Inducible	Upregulated during inflammation; may enhance viral adhesion and local immune activation without productive replication; potential role in placental immune activation and targeted anti-inflammatory therapy.	(79)

Following initial receptor-mediated entry, many viruses actively modulate host cell receptor expression through both direct transcriptional regulation and indirect activation of inflammatory pathways. For example, CMV infection engages β1 and αvβ3 integrins via viral glycoproteins (gB/gH) to mediate entry and downstream signaling, sustaining chronic infection (47). After receptor-mediated endocytosis, viruses like HSV-1/HSV-2 can establish latent infections in cellular compartments while simultaneously promoting receptor recycling to the cell surface, ensuring continued viral entry capacity (48, 49). This mechanism enables viral persistence by maintaining portal availability while avoiding complete receptor depletion, which would limit the potential for reinfection.

During trophoblast maturation from cytotrophoblastic precursors to multinucleated syncytiotrophoblastic entities, these cells undergo substantial alterations in cell surface receptor repertoires and expression profiles (48–51). Persistent viral pathogens exploit these developmental transitions through sophisticated adaptive mechanisms, including receptor tropism switching to engage newly upregulated cellular entry molecules during differentiation processes, multi-receptor utilization strategies that employ redundant entry pathways to maintain infectivity despite dynamic receptor expression patterns, and establishment of compartmentalized viral persistence within discrete trophoblast subpopulations that exhibit stable receptor expression phenotypes. Additionally, tissue-resident and migratory immune cell populations, particularly dendritic cells and macrophages that traverse the maternal-fetal interface, maintain constitutive receptor expression profiles that facilitate sustained viral maintenance across anatomical compartments (52). The extended lifespan and inherent migratory properties of these professional antigen-presenting cells create mobile viral reservoir systems capable of disseminating infection to naive cellular targets and establishing secondary infection foci throughout the maternal-fetal interface, thereby perpetuating viral persistence despite host immune surveillance and tissue-specific antiviral responses. Persistent viruses actively manipulate host receptor expression to maintain chronic infections (23, 47, 53–55).Viral proteins directly interact with host transcription factors to upregulate specific receptors (e.g., CMV immediate early proteins enhancing integrin expression). Persistent viruses induce chromatin modifications that maintain elevated receptor expression even during periods of viral dormancy. Viral miRNAs target cellular miRNAs that normally downregulate viral entry receptors, creating stable high-expression states.

The unique architecture of syncytiotrophoblasts facilitates viral persistence independently of continued receptor-mediated entry. Once established within the syncytium, viruses can spread through cytoplasmic continuity, thereby reducing their dependence on receptor availability while maintaining a persistent infection. However, receptor expression remains critical for initial syncytial establishment, requiring receptor-mediated entry into the cytotrophoblast precursor as well as enabling viral spread to newly forming syncytial regions. The immunologically privileged nature of the maternal-fetal interface, combined with stable receptor expression, creates ideal conditions for viral persistence. Reduced immune surveillance allows infected cells to maintain viral reservoirs without elimination, while continued receptor availability ensures viral replication capacity.

### 3.2 Placental cell type susceptibility for viral infection

Cytotrophoblasts, the mononuclear progenitors that underlie and fuse to replenish the syncytiotrophoblast, express a heterogeneous repertoire of viral receptors (ACE2, AXL, integrins) and display gestational-age–dependent transcriptional programs that influence permissivity (27, 56). Receptor expression alone does not ensure productive replication: outcome depends on intracellular host factors, innate antiviral signaling (type I/III IFNs, ISGs), and metabolic state. Nonetheless, multiple ex vivo and primary-cell studies demonstrate cytotrophoblast permissiveness to selected pathogens (57, 58). First- and mid-trimester cytotrophoblast isolates and placental explants support ZIKV replication with release of infectious virus measured by focus-forming assays (FFA) and plaque-equivalent readouts, indicating bona fide productive infection in these progenitors (59, 60). By contrast, evidence for productive SARS-CoV-2 replication in cytotrophoblasts is inconsistent; ACE2/TMPRSS2 co-expression can permit viral entry, but many studies report limited or abortive replication despite detectable viral RNA or protein, suggesting post-entry restriction in cytotrophoblasts (61). CMV replicates in cytotrophoblasts in explant models with detection of viral immediate-early and late proteins and release of infectious particles, consistent with cytotrophoblasts acting as potential reservoirs for sustained placental infection. These data collectively indicate that cytotrophoblasts are variably permissive in a pathogen- and gestational-stage–dependent manner, and that demonstrating infectious-virus production (PFU/FFA/plaque equivalents) is critical to distinguish accurate replication from mere viral entry or cargo (47, 57).

AXL, a TAM-family receptor highly expressed during early and mid-gestation, mediates ZIKV entry via receptor-mediated endocytosis, with productive infection demonstrated in syncytiotrophoblasts, cytotrophoblasts, and Hofbauer cells; redundancy with other TAM receptors, however, indicates that AXL is not strictly essential for placental permissiveness (62–64). Beyond virus-specific receptors, integrins such as αvβ3, αvβ5, and αvβ6, which are broadly expressed on trophoblasts, support viral attachment and internalization for pathogens including CMV, adenoviruses, and enteroviruses. CMV binding to αvβ3 integrin is associated with productive placental infection, whereas adenoviral replication appears cell-type dependent (23). Collectively, these findings highlight that while receptor expression is necessary for viral entry, it does not invariably guarantee productive replication or vertical transmission, emphasizing the complex interplay of viral, cellular, and gestational factors that govern placental susceptibility.

Hofbauer cells, the fetal villous macrophages, are antigenically and functionally heterogeneous but are repeatedly shown to be highly permissive to a range of viruses and to support productive replication. Multiple primary-cell and explant studies have demonstrated robust replication of ZIKV in isolated Hofbauer cells with release of infectious virions quantified by FFA (reporting increases in infectious titer over time following infection at defined MOIs), and immunohistochemical detection of viral proteins in Hofbauer cells within infected villous explants confirms in-tissue permissivity (17, 65). Similarly, CMV productively infects placental macrophages *in vitro* and *in situ*, with increases in infectious-virus readouts and upregulation of proinflammatory mediators that can modulate local permissivity (66). HIV-1 can infect and be amplified by Hofbauer cells. These cells support viral replication and act as local reservoirs, although replicative efficiency varies with viral strain and co-infections (e.g., CMV can enhance HIV replication in Hofbauer cells) (67, 68). The strength of this evidence is supported by quantitative infectious-virus assays (FFA/PFU equivalents) and by detection of replicative intermediates in HBCs, underscoring their role as amplification hubs at the maternal–fetal interface.

### 3.3 Immunological microenvironment in placenta

The placental immune environment is a unique area that needs to balance the acceptance of the semi-allogeneic fetus with protection against infections. This careful balance is maintained through complex interactions among maternal immune cells, placental cells, and fetal cells. As a result, this environment may be especially vulnerable to certain viral infections (52).Maternal immune cells, including decidual macrophages, natural killer cells, and T cell populations, infiltrate placental tissues and contribute to both protective immunity and immunopathology during viral infections. These cells express various pattern recognition receptors that can detect viral components and initiate antiviral responses, but their activation must be carefully regulated to prevent rejection of the fetus (52). Placental trophoblasts themselves contribute to this immunological balance by expressing immunomodulatory molecules such as HLA-G, transforming growth factor-β, and anti-inflammatory cytokines. Viral infections can disrupt this equilibrium by altering cytokine and chemokine production, thereby tipping the balance toward pathology (52, 80).

Dendritic cells play a dual role in this setting. They express viral receptors, including DC-SIGN (CD209), AXL, and Tyro3, making them susceptible to ZIKV uptake (21). Their migratory capacity allows infected dendritic cells to traffic within decidual and placental tissues, potentially serving as “Trojan horses” that deliver the virus across the maternal–fetal interface. However, it is not established that these same dendritic cells migrate into the fetal brain. Instead, current evidence indicates that once ZIKV crosses the placental barrier, the virus itself gains access to the fetal circulation, where it can infect fetal endothelial and neural progenitor cells, explaining the well- recognized neurotropism and congenital brain pathology (81, 82). Thus, the primary role of dendritic cells is to mediate initial viral translocation at the placental interface, rather than to directly disseminate to fetal organs.

### 3.4 Gestational age-related changes in receptor expression

Placental receptor expression undergoes dramatic temporal modulation throughout pregnancy, creating windows of differential susceptibility to viral infection. These changes reflect the evolving functional demands of the placenta, from initial implantation and establishment of maternal-fetal circulation to mature metabolic exchange and preparation for parturition. Understanding these temporal patterns is crucial for predicting viral transmission risk and developing gestational age-specific prevention strategies. During early pregnancy, placental viral receptor expression shows unique traits, including increased AXL expression. The highest level of syncytiotrophoblast in the first trimester is linked to a greater vulnerability to the Zika virus (36, 59). However, low ACE2 expression in early pregnancy may offer some protection against SARS-CoV-2, and this expression and associated risk rise as pregnancy progresses. High CD46 levels in early cytotrophoblasts are crucial for both placental development and vulnerability to viruses, esp. CMV (83). Hormonal changes in early pregnancy often regulate this.

#### 3.4.1 Temporal changes in the expression of viral entry receptor (6–12 weeks)

##### 3.4.1.1 ACE2 and TMPRSS2 temporal dynamics

ACE2 expression reaches maximum levels in first-trimester syncytiotrophoblasts and cytotrophoblasts, declining progressively through the second and third trimesters (69, 72, 74). Parallel expression pattern with ACE2, highest in early pregnancy when trophoblast proliferation and invasion are maximal (73, 84).Estradiol and progesterone influence ACE2/TMPRSS2 expression through estrogen and progesterone response elements (85).

##### 3.4.1.2 Integrin expression modulation

Dramatic upregulation of αvβ3 Integrin during implantation window (6–8 weeks), facilitating both embryo attachment and CMV susceptibility (71).Progressive increase in α1β1 and α2β1 integrins throughout the first trimester, supporting trophoblast invasion and creating enhanced viral entry opportunities (86). Integrin expression changes correlate with decidual remodeling and viral accessibility (87).

##### 3.4.1.3 Growth factor receptor dynamics

Highest expression of PDGFR in early pregnancy trophoblasts, serving as CMV co-receptor (88). Further, ErbB1–4 receptor expression peaks in the first trimester, potentially facilitating viral exploitation of growth signaling (89).

#### 3.4.2 Second trimester receptor transitions (13–26 weeks)

##### 3.4.2.1 AXL receptor family modulation

Maximum AXL expression occurs in mid-pregnancy syncytiotrophoblasts, correlating with highest Zika virus susceptibility window (70).TAM receptor family members show coordinated expression patterns of Tyro3 and Mer co-expression affecting flavivirus susceptibility (90).Phosphatidylserine-binding proteins that bridge viral particles to AXL receptors show gestational regulation (70).

##### 3.4.2.2 Complement regulatory protein changes

Decay-accelerating factor and membrane cofactor protein expression increase progressively, affecting viral susceptibility patterns (91) with local complement regulation affecting viral-complement interactions (92).

##### 3.4.2.3 Fc receptor expression dynamics

Progressive upregulation of FcRn (Neonatal Fc Receptor) throughout the second trimester for IgG transport, potentially affecting antibody-dependent viral enhancement (93). Gestational regulation of FcγRII and FcγRIII in Hofbauer cells affecting dengue virus antibody-dependent enhancement (94).

#### 3.4.3 Third trimester receptor evolution (27–40 weeks)

Significant downregulation of ACE2/TMPRSS2 in the mature placenta, potentially explaining lower SARS-CoV-2 transmission rates in late pregnancy (69). Also a shift from invasive (αvβ3, α5β1) to maintenance integrins (α6β4, α3β1) affects viral entry patterns (95). Further, TLR2, TLR4, and TLR9 upregulation in late pregnancy, create enhanced viral recognition but also potential exploitation (96). Cytokine receptors, IL-1R, TNF-R, and interferon receptors show gestational regulation affecting antiviral responses (96). Progressive upregulation of ICAM-1 and VCAM-1 in placental endothelium throughout pregnancy, enhancing susceptibility to rhinoviruses and other pathogens (97).

#### 3.4.4 Epigenetic regulation of temporal viral receptor expression

Gestational changes in receptor gene promoter methylation affecting expression (98) with Placenta-specific methylation patterns distinct from maternal tissues (99). Active and repressive histone marks show gestational dynamics of H3K4me3 and H3K27me3 at receptor loci (100, 101). Further, pregnancy-specific miRNAs, C19MC and C14MC miRNA clusters affects receptor expression (102, 103). The Chromosome 19 miRNA Cluster (C19MC) and the Chromosome 14 miRNA Cluster (C14MC) are extensive collections of microRNAs (miRNAs) that are mainly found in the human placenta and are essential for pregnancy. C19MC is an imprinted gene exclusive to primates. It is expressed from the paternal allele, whereas C14MC is found in eutherian mammals and is expressed from the maternal allele, both of which are located within imprinted genomic regions. In primary trophoblastic cells, C19MC miRNA are active against ZIKV, through IFN lambda-1-mediated resistance (104).

### 3.5 Hormonal regulation of viral receptor expression

Pregnancy is characterized by profound endocrine adaptations that modulate viral receptor expression in the placenta, thereby dynamically shaping susceptibility to infection. Estrogens, particularly estradiol (E2) and estriol (E3), exert transcriptional control over a wide range of viral entry factors, including angiotensin-converting enzyme 2 (ACE2), integrins, and select growth factor receptors, through estrogen response elements within promoter regions (8, 85). While estradiol enhances ACE2 and integrin expression in endothelial and trophoblastic compartments, estriol, unique to pregnancy, reshapes placental receptor landscapes, fine-tuning maternal–fetal immunotolerance and transport functions (105, 106). Progesterone further exerts a dual regulatory effect, broadly suppressing inflammatory receptor pathways while preserving structural and adhesion-related receptor pools critical for placental integrity (107, 108). In addition to steroid hormones, glycoprotein hormones such as human chorionic gonadotropin (hCG) modulate receptor expression via cAMP-dependent pathways, influencing trophoblast differentiation and receptor availability at the syncytiotrophoblast surface (109). Similarly, human placental lactogen (hPL), through its metabolic and growth-promoting actions, alters growth factor receptor signaling in placental and fetal tissues, thereby indirectly impacting viral entry pathways (9). Together, these hormonal axes create a temporally dynamic receptor profile across gestation, wherein the relative balance of steroidal and peptide hormones not only governs placental physiology but also imposes gestational windows of heightened or reduced vulnerability to viral exploitation.

Gestational age and hormone–dependent susceptibility represents a conceptual framework in which viral entry factors, placental physiology, and maternal immune tolerance intersect to create temporally defined “windows of vulnerability” for vertical transmission. Prior reviews have often catalogued receptor expression patterns without fully interrogating their implications for infection risk (74, 110). The following advances this by situating receptor dynamics within a temporal hierarchy of susceptibility. For example, high AXL expression in the first trimester corresponds to the well-documented vulnerability of early pregnancies to ZIKV (17), whereas low ACE2 expression during the same period may confer relative protection against SARS-CoV-2, only for susceptibility to rise again in the third trimester with ACE2 upregulation (20). This duality underscores that no virus exploits gestational age uniformly; rather, each pathogen operates within its own “susceptibility window,” shaped by receptor availability, placental remodeling, and maternal systemic physiology.

Further, susceptibility windows are not rigid, but plastic. Hormonal regulation (through estrogen, progesterone, and hCG signaling) fine-tunes receptor landscapes across trimesters (9, 105, 106, 109). Additionally, epigenetic regulation, such as C19MC miRNAs known to restrict viral replication creates trimester-specific antiviral states (9). This may explain why certain viruses, like CMV, appear capable of transmission throughout gestation but manifest distinct fetal phenotypes depending on timing: sensorineural deficits are often linked to first-trimester transmission, while growth restriction is more associated with later infections (111).

### 3.6 Cytokine regulation of placental virus receptors

The placental microenvironment is intricately regulated by cytokines, which modulate the expression of viral entry receptors, thereby influencing maternal-fetal transmission risks. Pro-inflammatory cytokines such as tumor necrosis factor-alpha (TNF-α), interleukin-1 beta (IL-1β), and interferon-gamma (IFN-γ) upregulate receptors like ACE2, CD46, and heparan sulfate proteoglycans on trophoblast and decidual cells, enhancing susceptibility to viruses including SARS-CoV-2, cytomegalovirus, and Zika virus (112, 113). Conversely, anti-inflammatory factors like interleukin-10 (IL-10), transforming growth factor-beta (TGF-β), and progesterone-induced regulatory elements downregulate these receptors and promote antiviral restriction factors, establishing protective barriers against viral entry (114). The dynamic shift from a pro-inflammatory state in early pregnancy to an anti-inflammatory one in the second trimester correlates with changes in viral receptor expression and periods of varying susceptibility (114). Clinically, profiling cytokine levels and viral receptor expression can serve as predictive biomarkers for maternal-fetal transmission risks, aiding in risk stratification during viral outbreaks (115).

Viral receptors in the placenta are key factors in how viruses move from mother to fetus. They are important targets for treatment. The patterns of receptor expression vary across different types of placental cells. Hormonal and inflammatory signals regulate these patterns, and they change throughout pregnancy. This creates a changing landscape for viral susceptibility. To develop effective ways to prevent congenital infections and safeguard the health of mothers and babies, it is essential to understand these mechanisms. New technologies, like single-cell analysis and spatial transcriptomics, are offering valuable insights into the biology of viral receptors in the placenta. These developments, along with a better understanding of how receptors work and are regulated, are creating new opportunities for diagnosis and treatment. As we deal with new viral threats during pregnancy, knowledge of placental viral receptors will be crucial for protecting the health of mothers and their children.

### 3.7 Mechanisms of virus transmission

#### 3.7.1 Transcytosis and paracellular transport

**Figure 1:**
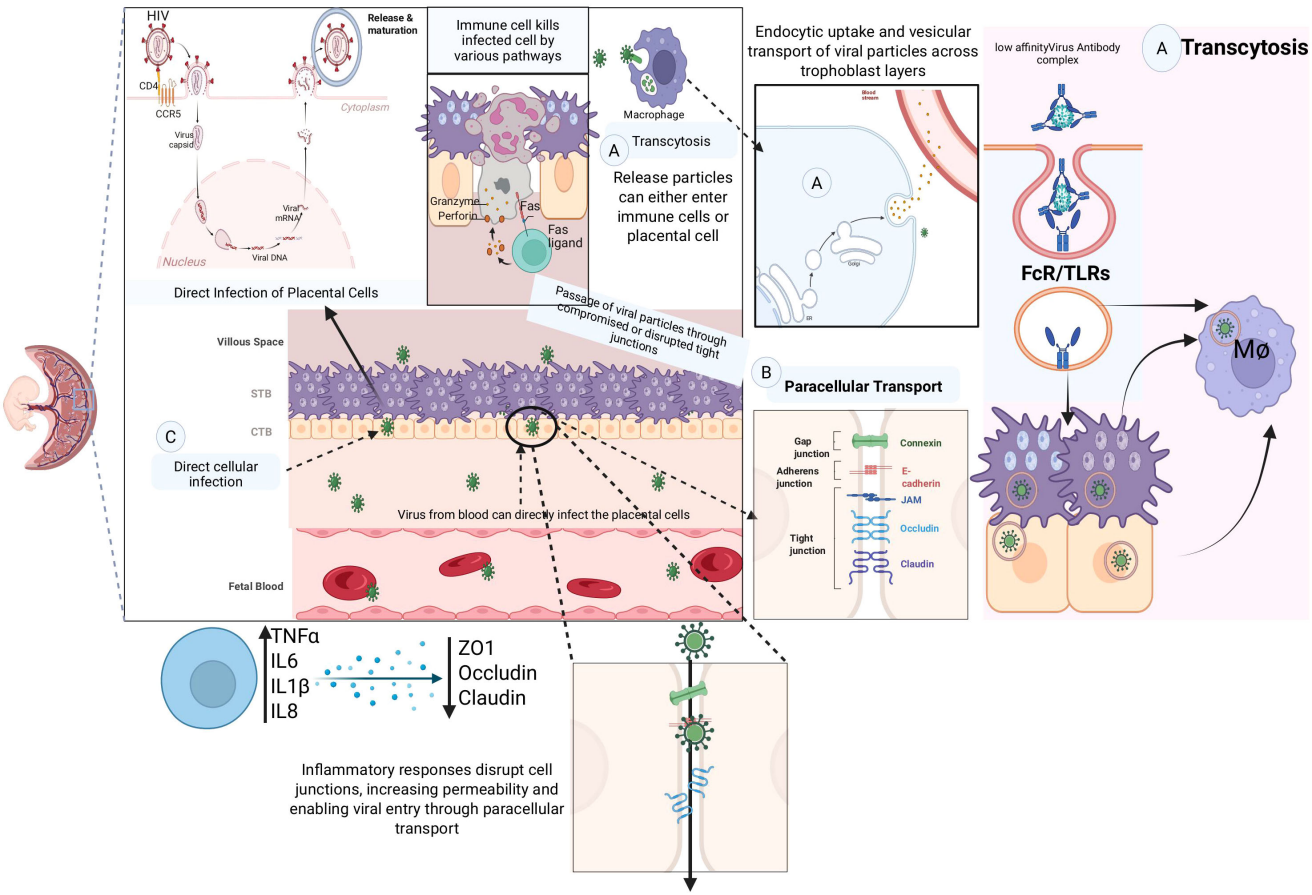
Placental architecture and viral transmission pathways.

Viral reservoirs within placental tissues represent critical determinants of vertical transmission efficiency and persistence. These reservoirs are established through multiple mechanisms, including direct cell-to-cell spread, creation of persistently infected cell populations, and exploitation of immune-privileged niches. Understanding reservoir dynamics is essential for developing therapeutic strategies to prevent maternal-fetal transmission. Viruses can cross placental barriers through transcytosis and paracellular transport mechanisms (**Figure 1**). Transcytosis represents a sophisticated cellular transport mechanism enabling macromolecular transfer across polarized epithelial barriers. In the placental context, transcytosis occurs primarily through three distinct pathways: receptor-mediated endocytosis, fluid-phase pinocytosis, and caveolae-mediated transport. Each pathway offers unique opportunities for viral exploitation while serving essential physiological functions. From an infection point of view, it involves the vesicular transport of viral particles across cells without productive infection, allowing viruses to reach the fetal circulation without necessarily establishing placental infection (116, 117).The syncytiotrophoblast exhibits active transcytotic transport for various substances, and some viruses may exploit these pathways for maternal-fetal transmission. However, the efficiency of viral transcytosis varies significantly between different viral species and may be influenced by viral particle size, surface properties, and interactions with cellular transport machinery (117, 118).Neonatal Fc receptor (FcRn) binds maternal IgG at acidic pH (6.0-6.5) in early endosomes, protecting antibodies from lysosomal degradation and facilitating transcytosis to fetal circulation (93). Immune complexes formed between maternal antibodies and viral particles can be co-transported via FcRn-mediated transcytosis, potentially facilitating viral transfer while evading immune recognition (93). Studies using fluorescently-labeled HIV-antibody complexes demonstrate transcytosis across trophoblast monolayers with 15-20% transport efficiency over 4 hours, significantly higher than virus alone (2-3%) (119). Transferrin receptor (TfR1) facilitates iron delivery but can be hijacked by viruses through molecular mimicry or direct binding (120). Caveolar invaginations facilitate transcytosis of lipid-associated molecules and can transport enveloped viruses (121). Paracellular transport occurs when viral particles pass between cells through compromised tight junctions or other intercellular connections. Viral infections and associated inflammatory responses can compromise the integrity of the placental barrier, creating pathways for enhanced viral transmission and the entry of different potentially harmful substances (117, 118).

Tight junctions consist of transmembrane proteins (claudins, occludins, JAMs) linked to cytoplasmic scaffolding proteins (ZO-1, ZO-2, ZO-3) that regulate paracellular permeability (122). SARS-CoV-2 spike protein binding to ACE2 triggers downstream signaling that affects tight junction protein phosphorylation and localization (123, 124). Treatment of polarized trophoblast cultures with SARS-CoV-2 spike protein causes 30-50% reduction in ZO-1 and claudin-1 immunofluorescence intensity at cell borders within 6–12 hours (72). Zika and Dengue viruses activate MMP-2 and MMP-9, causing proteolytic cleavage of tight junction proteins (125).

#### 3.7.2 Direct cell-to-cell spread mechanisms

The syncytiotrophoblast’s multinucleated architecture enables viral spread through cytoplasmic continuity without cell membrane barriers (126). Membranous connections between non-adjacent cells facilitate viral transfer while avoiding extracellular immune surveillance (127).Gap junctions formed by connexin proteins can facilitate the transfer of small viral components and signaling molecules (128). Viruses can induce the formation of specialized cell-cell contacts resembling immunological synapses for efficient transmission. Human T-lymphotropic virus type 1 creates specialized synapses with high transmission efficiency compared to the cell-free virus (129).

#### 3.7.3 Long-term viral reservoir establishment

Hofbauer cell dynamics help as a reservoir to persist throughout pregnancy, creating stable viral reservoirs (130). Cytotrophoblast stem cells maintain self-renewal capacity and can harbor persistent viral infections. Studies using primary cytotrophoblast stem cell cultures show CMV can establish latent infections with minimal viral gene expression, reactivating upon differentiation signals (33).Placental endothelial cells form extensive networks, enabling viral dissemination throughout fetal circulation. Experimental infection of placental endothelial cell cultures demonstrates that HSV-1 can establish persistent infections with periodic shedding cycles (113, 131). This type of spread can lead to the formation of viral reservoirs within placental tissues that may persist throughout pregnancy and serve as sources of ongoing fetal exposure (27). Going by this logic, it seems conceivable that syncytiotrophoblast cells are better transmitters of the virus as compared to mononucleated cytotrophoblasts.

This schematic illustrates the structural organization of the human placenta and mechanistic routes by which viruses may breach the maternal–fetal barrier. Maternal blood within the intervillous space carries pathogenic viral particles that encounter sequential placental layers including the decidua, maternal endothelium, syncytiotrophoblast (STB), cytotrophoblast (CTB), villous stroma, and fetal capillary endothelium. Three principal transmission pathways are highlighted: (i) Direct cellular infection – Viral particles directly infect STBs and penetrate into underlying CTBs, enabling access to villous stromal compartments and fetal capillaries. Productive infection at this level compromises trophoblast integrity, alters syncytial turnover, and facilitates vertical transmission. (ii) Transcytosis – Endocytic uptake and vesicular transport shuttle viral particles across trophoblasts without productive replication. Fc receptor (FcR)– and Toll-like receptor (TLR)-mediated trafficking of virus–antibody complexes to Hofbauer cells and macrophages illustrates how viruses exploit placental immune regulatory pathways to cross into fetal circulation while simultaneously avoiding neutralization. (iii) Paracellular transport – Viral dissemination occurs through compromised intercellular junctions. Inflammatory cytokines (TNFα, IL-6, IL-1β, IL-8) downregulate tight junction proteins (ZO-1, occludin, claudin) and adherens junction molecules, thereby increasing epithelial permeability. This cytokine- driven junctional remodeling permits paracellular viral passage, linking infection to inflammation-induced barrier dysfunction. In parallel, decidual and villous immune cells contribute to the pathophysiology: neutrophils and macrophages mount antiviral responses but also generate pro-inflammatory cytokines that exacerbate junctional disruption and vascular permeability; Hofbauer cells act as both viral reservoirs and amplifiers of inflammatory signaling. These converging processes depict how viral infection, immune activation, and barrier compromise integrate to facilitate transplacental transmission and heighten the risk of adverse pregnancy outcomes, including fetal growth restriction, stillbirth, and neurodevelopmental disorders.

## 4 Placental immune responses to viral infections

### 4.1 Innate immune recognition and signaling

**Figure 2:**
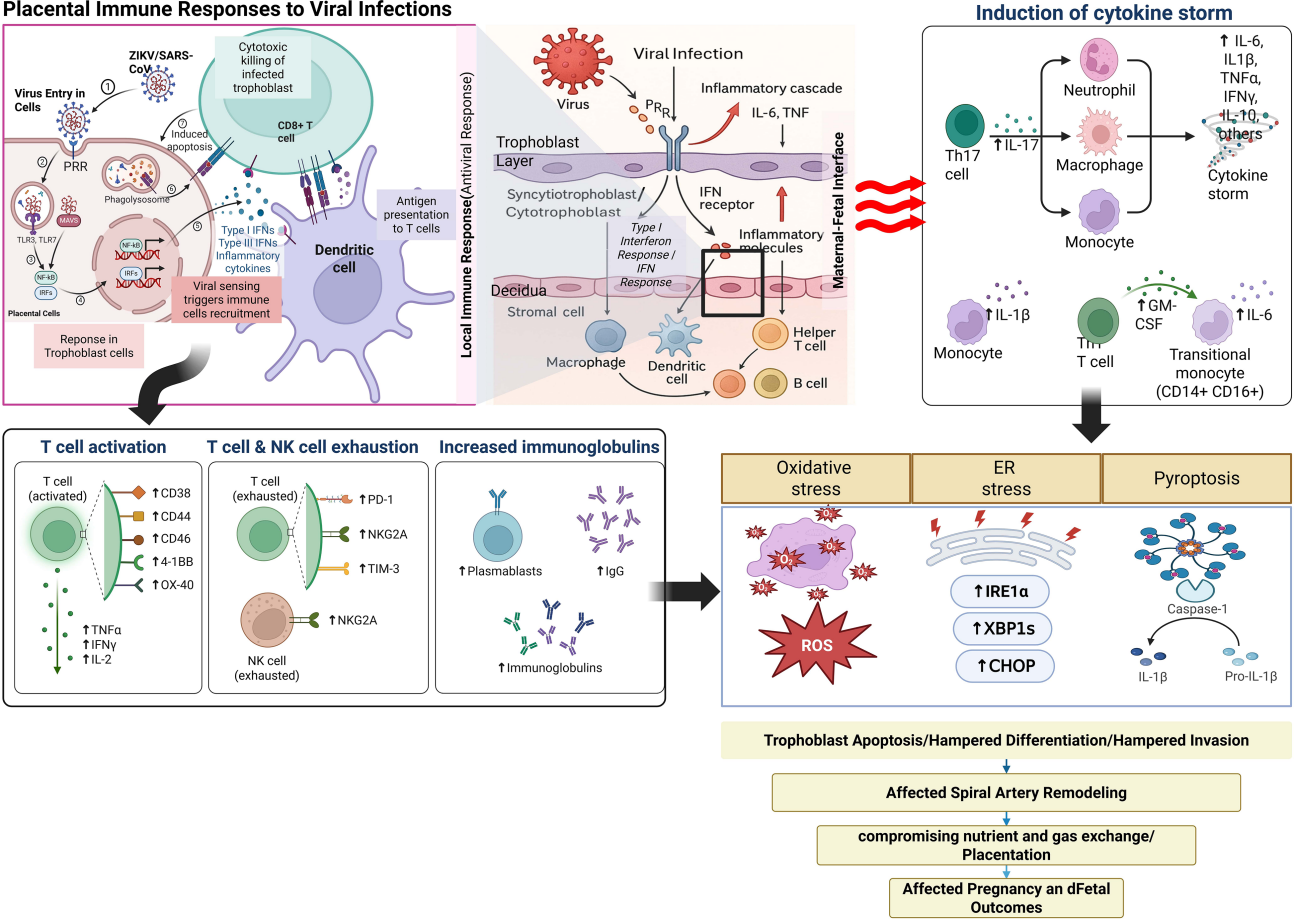
Immune responses to viral infections in placental tissues.

The placenta expresses a comprehensive array of pattern recognition receptors (PRRs) that detect viral components and initiate antiviral immune responses (**Figure 2**). Toll-like receptors (TLRs), RIG-I-like receptors (RLRs), and cGAS-STING pathways all contribute to viral recognition and downstream signaling in placental cells (132, 133).TLR3, TLR7, TLR8, and TLR9 recognize viral nucleic acids and trigger interferon responses in placental cells (134). However, the expression and functional activity of these receptors vary between different placental cell types and gestational ages, creating temporal and spatial heterogeneity in antiviral responses. The syncytiotrophoblast, in particular, exhibits unique patterns of TLR expression that may influence its susceptibility to different viral pathogens (56).

The cGAS-STING pathway has emerged as a critical antiviral defense mechanism in placental cells, particularly for DNA viruses and retroviruses (135). This pathway detects cytoplasmic DNA and triggers robust interferon responses, but excessive activation can lead to inflammatory damage that compromises placental function (136). Understanding the regulation of cGAS-STING signaling in placental cells is crucial for developing therapeutic approaches that enhance antiviral immunity without causing adverse effects on the placenta.

#### 4.1.1 Toll-like receptor viral recognition

All 10 Toll-like receptors (TLRs) are expressed in the human placenta, along with adapter proteins such as MyD88, MD-2, TRIF, TIRAP, and TRAM, and accessory molecules like CD14 (137). This broad receptor–adapter repertoire equips the placenta to detect and respond to a wide variety of pathogens. While not exclusively virus-specific, certain TLRs play a particularly prominent role in antiviral defense. For example, TLR3 recognizes viral double-stranded RNA and signals through the TRIF-dependent pathway to induce type I interferon responses (138). Similarly, TLR7 and TLR8 detect single-stranded RNA and activate antiviral pathways, as shown in Hofbauer cells, thereby contributing to defense against RNA viruses such as SARS-CoV-2, Zika virus, and influenza viruses (139). This innate immune recognition pathway serves as a fundamental surveillance system for RNA viral pathogens, demonstrating activity against SARS-CoV-2, Zika virus, and influenza viruses. TLR9 functions through cytosine-guanine dinucleotide (‘CpG’ motif) recognition to initiate cellular antimicrobial responses against bacterial DNA while also mediating immune activation upon exposure to DNA viruses, including herpes virus family members and cytomegalovirus (140).

Beyond TLR-mediated recognition, placental trophoblasts constitutively secrete type III interferons (IFN-λ1 and IFN-λ2), which act in both paracrine and autocrine fashions to restrict viral infections. Trophoblast organoids demonstrate detectable IFN-λ2 release by around 10 days post-plating (141). ISGs subsequently modulate immune cell functions, with trophoblast-derived PD-L1 promoting macrophage M2 polarization to balance antiviral defense with immune tolerance. Autophagy also operates as an antiviral mechanism at the maternal–fetal interface (142), while placental microRNAs fine-tune immune defenses through regulation of autophagy and interferon signaling (118). Additionally, NF-κB signaling is tightly regulated, providing antimicrobial protection while maintaining immune homeostasis in the placenta.

TLR2 recognizes HSV glycoproteins gB and gH/gL, triggering MyD88-dependent NF-κB activation in placental macrophages and trophoblasts (143). It also detects CMV envelope glycoproteins and tegument proteins, initiating pro-inflammatory responses in Hofbauer cells and decidual macrophages (143, 144).TLR3 recognizes viral dsRNA intermediates during replication in infected trophoblasts, triggering IRF3-mediated type I interferon responses. TLR3-mediated recognition of ZIKV dsRNA in placental cells initiates antiviral responses but can also contribute to inflammatory damage. TLR3-deficient BeWo cells demonstrate lower IFN-β production following poly(I:C) stimulation or ZIKV infection (145).TLR4 recognizes RSV F protein, triggering robust inflammatory responses in placental tissues (146). TLR4 detects influenza hemagglutinin and other viral proteins, initiating innate responses in placental immune cells (147).

#### 4.1.2 RIG-I-like receptor viral recognition

RIG-I recognizes influenza viral RNA with 5’-triphosphate modifications in infected trophoblasts and detects Sendai virus defective interfering particles, triggering robust IFN responses in placental cells (148, 149). MDA5 recognizes poliovirus dsRNA replication intermediates in infected cells. MDA5-dependent recognition triggers antiviral responses in placental tissues (148). LGP2 modulates RIG-I and MDA5 responses to HCV RNA. LGP2 fine-tunes antiviral responses to respiratory viral infections in placental cells (150, 151).

#### 4.1.3 cGAS-STING DNA sensing pathway

cGAS recognizes HSV DNA in infected trophoblasts, producing cGAMP and activating STING-dependent IFN responses (152). It also detects CMV DNA during nuclear replication, though viral proteins US9 and UL31 can inhibit cGAS activity (152). STING activation in response to HPV DNA triggers TBK1-IRF3 phosphorylation and IFN-β production in infected cervical and placental epithelia. STING-mediated responses to adenoviral DNA contribute to inflammatory pathology in placental tissues (153).

#### 4.1.4 NOD-like receptor viral recognition

NLRP3 recognizes influenza M2 ion channel activity and viral RNA, triggering IL-1β and IL-18 production in macrophages (154). NLRP3 activation by viral proteins and cellular stress leads to pyroptotic cell death in infected trophoblasts (155).NLRC5 regulates MHC-I expression in response to viral infections, affecting antigen presentation in placental cells (156). Several viruses (CMV, HRV, adenovirus) target NLRC5 function to evade immune recognition (156).

#### 4.1.5 Complement system viral recognition

The placental antiviral complement system plays a vital role as an innate immune defense mechanism, offering wide-ranging protection against viral infections at the maternal-fetal interface by activating classical, alternative, and lectin pathways in a coordinated manner. Trophoblast cells produce complement components such as C1q, C3, and factor B, allowing for local synthesis and activation of the complement system in response to viral threats, while also generating complement regulatory proteins like CD55 (decay-accelerating factor), CD46 (membrane cofactor protein), and CD59 (protectin) to prevent damage from excessive complement activity (157). The system operates through various antiviral strategies, including direct neutralization of viruses through C3b opsonization, facilitating the clearance of viruses via phagocytosis by placental macrophages mediated by complement receptors, and enhancing adaptive immune responses through the recruitment and activation of immune cells by C3a and C5a. The activation of complement in the placenta shows regulation that is specific to different stages of gestation, with increased activity during early pregnancy when the risk of viral infections is higher, followed by a controlled decrease to promote maternal-fetal tolerance while maintaining antiviral function (158, 159). Any dysregulation in placental complement activity, whether caused by genetic defects, maternal autoimmune disorders, or strategies employed by pathogens to evade the complement system, significantly raises the risk of vertical transmission for various viral pathogens, underscoring the system’s essential role in safeguarding fetal development from complications caused by viruses.

### 4.2 Placental interferon-stimulated gene system

The placental tissue exhibits sophisticated ISG expression patterns, which create a robust antiviral state while maintaining essential maternal-fetal tolerance. These ISGs exhibit cell type-specific expression profiles and gestational age-dependent regulation, reflecting the unique immunological requirements of the maternal-fetal interface. ISG20 is an interferon-inducible 3′–5′ exonuclease with potent antiviral activity against a range of RNA viruses. In trophoblast cells, ISG20 plays a critical role in restricting ZIKV replication by directly degrading viral RNA. Studies have shown that ISG20 induction following type I interferon signaling leads to selective cleavage of ZIKV genomic RNA, thereby preventing accumulation of viral proteins and blocking productive infection. Importantly, engineered recombinant ISG20-Fc fusion proteins not only exhibit enhanced stability and antiviral efficacy but also significantly reduce ZIKV replication in both *in vitro* trophoblast cultures and *in vivo* mouse models of congenital infection (160). Upon TLR3 activation (e.g., by viral double-stranded RNA intermediates or the synthetic analog poly(I:C)), trophoblasts mount a strong antiviral response driven by IFN-β secretion. IFN-β not only acts in an autocrine manner to reinforce antiviral defenses but also engages in a positive feedback loop amplifying the expression of downstream SGs (161). Among these classical ISGs are Secretory Leukocyte Protease Inhibitor (SLPI): contributes to mucosal and placental barrier defense, exhibiting antiviral and anti-inflammatory functions (162). 2′,5′-Oligoadenylate Synthetase (OAS) that activates RNase L to degrade viral RNA (163). MxA (Myxovirus Resistance Protein A), a GTPase that interferes with viral nucleocapsid transport and replication (164). APOBEC3G a cytidine deaminase that restricts retroviral replication by inducing hypermutation in viral genomes (165). Type I interferons (IFN-α/β) represent the primary antiviral effector mechanism in placental cells, inducing the expression of hundreds of ISGs that create cellular antiviral states (166). However, the interferon response in placental cells exhibits unique characteristics that may influence the outcome of viral infections. The syncytiotrophoblast shows constitutive expression of several ISGs even in the absence of viral infection, suggesting a primed antiviral state that may protect against specific pathogens (166). However, this baseline activation may also create a refractory state that limits the ability to mount robust responses to new viral challenges. Type III interferons (IFN-λ) also play important roles in placental antiviral immunity, particularly at mucosal surfaces. These interferons may protect against viral infections while causing less inflammatory damage than type I interferons, making them attractive targets for therapeutic intervention (166). Together, these ISGs establish an intracellular antiviral state in trophoblasts, effectively limiting viral replication and spread at the maternal–fetal interface.

### 4.3 Adaptive immune responses

While the placenta is not a traditional secondary lymphoid organ, it does support various adaptive immune responses that contribute to antiviral immunity. Maternal antibodies cross the placenta through FcRn-mediated transport, providing passive immunity to the fetus but also potentially facilitating antibody-dependent enhancement of certain viral infections (167).T cell responses within placental tissues are complex and must be carefully regulated to maintain maternal-fetal tolerance while protecting against pathogens (113, 168). Beyond traditional antibody-mediated responses, cellular cytotoxicity plays a crucial role through multiple pathways: uterine natural killer (uNK) cells deploy perforin and granzyme-mediated cytotoxicity against virus-infected trophoblasts while simultaneously regulating placental vascularization (169), while CD8+ cytotoxic T lymphocytes recognize viral peptides presented on MHC Class I molecules to eliminate infected decidual and placental cells (170). The placental immune environment is further modulated by tissue-resident memory T cells (TRM) that provide rapid secondary responses to viral re-exposure, and by the delicate balance between pro-inflammatory Th1/Th17 responses that promote viral clearance and Tregs that maintain maternal-fetal tolerance (171). Viral infections can exploit or disrupt trophoblast-mediated immune suppression mechanisms, including reduced MHC Class I expression and immunosuppressive factor secretion, leading to altered decidual immune cell crosstalk and potentially compromising both placental barrier function and vertical transmission dynamics (27). This intricate network of adaptive immune mechanisms ultimately determines whether maternal antiviral responses protect or inadvertently harm fetal development through immune-mediated placental pathology.

This schematic integrates innate and adaptive immune responses within placental tissues upon viral infection and illustrates their pathological consequences. Innate sensing and antiviral signaling: Viral entry into trophoblasts activates pattern recognition receptors (PRRs), including TLR3 and TLR7, leading to recruitment of adaptor proteins (e.g., MAVS) and downstream activation of transcription factors (NF-κB, IRF3, IRF5). This cascade induces type I and type III interferons and inflammatory cytokines (IL-6, TNF-α, IL-1β, IL-8) that establish an antiviral state and recruit immune cells. Adaptive immune activation: Dendritic cells (DCs) phagocytose viral antigens and present them to CD8^+^ T cells, driving cytotoxicity and apoptosis of infected trophoblasts. In parallel, CD4^+^ helper T cells and B cells promote adaptive responses, including increased immunoglobulin production (IgG, plasmablast expansion). However, persistent stimulation leads to T cell and NK cell exhaustion, marked by upregulation of inhibitory receptors (PD-1, NKG2A, TIM-3), impairing cytotoxic clearance. Cytokine amplification and cytokine storm: Excessive activation of monocytes, neutrophils, macrophages, and Th17 cells elevates cytokines (IL-6, IL-1β, TNF-α, IFN-γ, GM-CSF), generating a cytokine storm that drives systemic and local placental inflammation. Cellular stress and damage pathways: Infected trophoblasts undergo oxidative stress (ROS accumulation), ER stress (↑IRE1α, XBP1s, CHOP), and pyroptosis (caspase-1 activation, IL-1β release). These stress pathways culminate in trophoblast apoptosis, impaired differentiation, and restricted invasion, disrupting spiral artery remodeling. Pregnancy consequences: The combined effects of immune hyperactivation, cellular stress, and barrier breakdown compromise nutrient and gas exchange at the maternal–fetal interface, leading to placental insufficiency and adverse pregnancy outcomes, including fetal growth restriction, preterm birth, and pregnancy loss.

### 4.4 Immune evasion mechanisms of virus

Pathogens have developed advanced techniques to bypass the immune defenses of the placenta, allowing them to infect the maternal-fetal interface successfully. These evasion tactics take advantage of both the natural immune tolerance present during pregnancy and the unique cellular structure of the placenta. Grasping these viral strategies is essential for developing targeted treatments and comprehending the complications that arise during pregnancy. Virus-encoded mechanisms frequently target type I interferon pathway components, with congenital pathogens including ZIKV, HIV, CMV, and SARS-CoV-2 known to downregulate this pathway (27). Herpes virus infection suppresses IFN-β in trophoblast cells, with viral-induced IFN-β inhibition resulting in robust inflammatory response to low bacterial concentrations and enhanced preterm birth risk, illustrating how immune evasion intersects with pathophysiological outcomes (172, 173). Viral ssRNA induces pro-inflammatory and type I interferon response in trophoblasts, with this inflammatory process indirectly inducing trophoblast apoptosis and compromising placental integrity (114). The NS5 protein of ZIKV associates with and targets STAT2 for destruction, thereby disrupting the JAK-STAT signaling pathway that typically promotes the transcription of interferon-stimulated genes (174). Multiple viruses downregulate MHC-I presentation to avoid CD8+ T cell recognition. Proteins like CMV US2/US11 target MHC-I heavy chains for proteasomal degradation (175). Viruses can upregulate host complement regulatory proteins to prevent complement-mediated lysis (176).Persistent viruses employ anti-apoptotic strategies to maintain infected cell viability (177). Maternal infection with SARS-CoV-2 leads to significantly reduced expression levels of essential innate immune mediators (IFNB, IFIT1, MXA, IL6, IL1B) in the chorionic villi and chorioamniotic membranes (113). CMV inhibits interferon-induced genes through various mechanisms and utilizes several genes that can independently reduce antigen presentation by downregulating MHC class I (178).

Building upon these individual mechanisms, viral immune evasion at the maternal–fetal interface can be broadly organized into three functional strategies: suppression of interferon-mediated antiviral signaling, modulation of antigen presentation and recognition, and exploitation of host immunoregulatory pathways. Suppression of interferon signaling represents a primary line of viral interference, as interferons are central to the placenta’s ability to mount rapid antiviral defenses. Beyond ZIKV-mediated STAT2 degradation (179), HCMV proteins like IE1 broadly perturbs host IFN pathways and surface proteins (180). SARS-CoV-2 similarly attenuates IRF3 activation in villous trophoblasts, thereby curtailing type I IFN release at the maternal–fetal interface (20). On a similar note, proteins like SKP2 suppresses autophagy (via BECN1) and interferon signaling (via IFNAR2), mechanisms not yet shown in placenta but likely relevant to viral immune evasion and other malignancies (181–183). By weakening this early innate alarm system, viruses create a permissive environment where replication can occur without immediate clearance.

The second major strategy involves modulation of antigen presentation. While CMV is the prototypical example, using US2 and US11 proteins to degrade classical MHC-I, it also actively shapes non-classical antigen presentation by increasing HLA-E and HLA-G expression (184, 185). This is significant because these molecules are naturally enriched in trophoblasts to promote maternal tolerance. By hijacking this system, CMV shields infected cells from both cytotoxic T lymphocytes and uterine NK cells, effectively mimicking pregnancy’s physiological tolerance mechanisms (186–188). These adaptations highlight how viruses co-opt placenta-specific immune features rather than simply suppressing them.

The third strategy is exploitation of host immunoregulatory pathways. Pregnancy is characterized by elevated levels of immune checkpoints, cytokines, and complement regulators that support fetal tolerance. Viruses capitalize on these features to extend their persistence. For instance, PD-L1 expression is naturally high in syncytiotrophoblasts in early and term placentas, helping maintain fetomaternal tolerance. Research shows syncytiotrophoblasts (but not cytotrophoblasts) strongly express PD-L1 in normal placenta (189). During infectious insults, PD-L1 expression is further elevated: a recent study found significantly enhanced PD-L1 in decidual stromal and extravillous trophoblast cells in placentas from women with acute or past SARS-CoV-2 infection compared to controls (190). Another immunoregulatory pathway is via anti-inflammatory cytokine IL-10. In HIV-infected mothers, placentas show higher IL-10 expression, especially in women receiving longer antiretroviral prophylaxis, compared to uninfected controls (189). IL-10 is known to suppress pro-inflammatory cytokine secretion and T cell activation; in other contexts, HIV-1 proteins (e.g. Nef) directly induce IL-10 in monocytes through signaling pathways involving calcium/calmodulin and p38 MAPK (191). Viruses also exploit dual-function receptors that serve both as immune regulators and viral entry points. A prime example is CD46 (membrane cofactor protein), which is both a complement regulatory protein and the receptor for measles virus. Multiple isoforms of CD46 have been shown to mediate measles virus binding and entry in cell models, indicating viruses can hijack a molecule normally protective against complement attack (192–194). Although data specifically in placental trophoblasts are sparse, the presence of CD46 in many human cells and its dual function suggest a plausible route for immune evasion in the placenta.

Together, these three strategies underscore how viruses do not simply bypass immunity, but rather integrate into the existing immunological architecture, including that of the placenta. By silencing interferon alarms, concealing themselves from adaptive immune recognition, and leveraging tolerance-promoting pathways, viruses achieve a balance between persistence and pathology. Understanding this interplay provides a clearer lens through which to design therapeutic interventions, such as IFN-boosting antivirals, checkpoint inhibitors adapted for pregnancy, or vaccines that prime protective immunity without disrupting tolerance.

## 5 Virus-specific mechanisms and pathogenesis

### 5.1 SARS-CoV-2 and COVID-19

**Figure 3:**
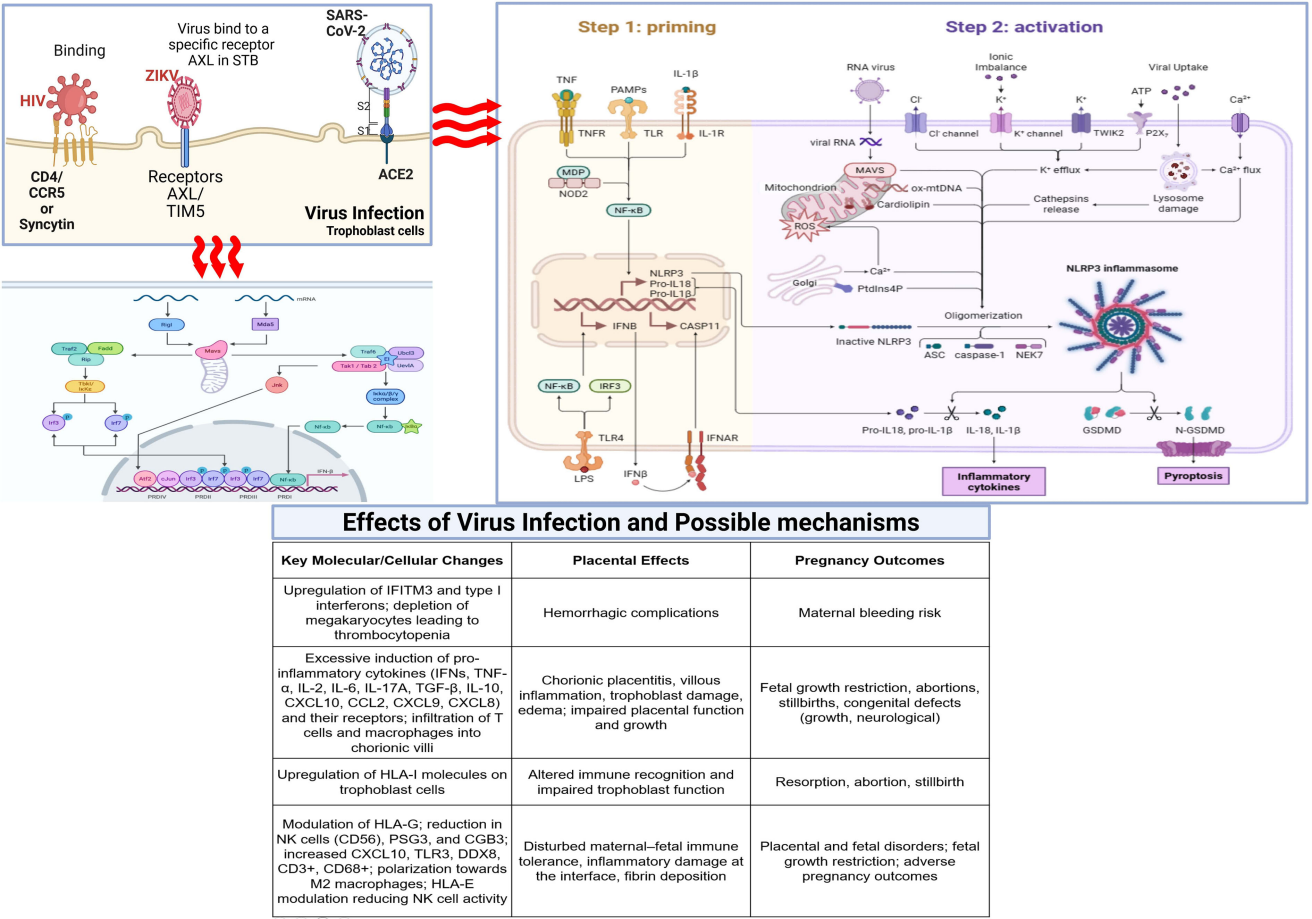
Virus-specific pathogenic mechanisms.

The COVID-19 pandemic has provided unprecedented insights into viral-placental interactions and their clinical consequences. SARS-CoV-2 exhibits variable placental tropism, with viral RNA detected in placental tissues in some, but not all, cases of maternal infection. The virus primarily targets cells expressing ACE2 and TMPRSS2, which are present in multiple placental cell types, including syncytiotrophoblast and decidual cells (8, 124).Placental pathology in COVID-19 cases includes maternal vascular malperfusion, increased fibrin deposition, and chronic histiocytic intervillositis (**Figure 3**) (195, 196). These findings suggest that placental damage may result from both direct viral effects and indirect consequences of maternal systemic inflammation. The relative contributions of these mechanisms likely vary depending on the timing of infection, viral load, and maternal immune status.

Maternal-fetal transmission of SARS-CoV-2 appears to be relatively uncommon compared to other respiratory viruses, but cases of confirmed congenital infection have been documented (196). The factors that determine transmission risk remain incompletely understood but may include gestational age at infection, severity of maternal disease, and individual variations in placental barrier function.

### 5.2 Zika virus and congenital Zika syndrome

Zika virus represents one of the most intensively studied examples of viral placental infection due to its association with severe congenital abnormalities, particularly microcephaly and other neurodevelopmental defects. The virus demonstrates strong tropism for placental cells, particularly those expressing the AXL receptor, which is highly expressed in trophoblasts and Hofbauer cells (27, 117).Zika virus infection leads to significant placental pathology, including trophoblast cell death, villous immaturity, and chronic villitis (**Figure 3**). These changes compromise placental function and may contribute to fetal growth restriction even in the absence of direct fetal infection (113). The virus can persist in placental tissues for extended periods, creating an ongoing risk for fetal transmission throughout pregnancy. While congenital Zika syndrome (CZS) represents a critical fetal outcome following maternal ZIKV infection, its detailed pathogenic mechanisms involving direct neurotropic viral invasion, disruption of neural progenitor cell proliferation, and induction of apoptosis in developing brain tissue fall beyond the scope of placental-focused discussions, as these neurological manifestations result from distinct viral mechanisms operating within fetal neural tissues rather than placental pathology (113). In the context of placental transmission, the primary concern centers on ZIKV’s capacity to breach the maternal-fetal barrier through infection of trophoblast cells, syncytiotrophoblast disruption, and exploitation of placental immune evasion mechanisms, thereby gaining access to fetal circulation and tissues (197). Importantly, ZIKV penetration of the placental barrier does not guarantee development of CZS in the exposed fetus, but rather establishes a high-risk scenario where fetal infection probability increases significantly, with actual syndrome development depending on multiple factors including gestational timing of maternal infection, viral load, maternal immune status, and fetal susceptibility factors (197). Therefore, while placental ZIKV infection represents a critical gateway event that enables fetal exposure and subsequent risk for severe developmental abnormalities including microcephaly, intracranial calcifications, and other neurological manifestations characteristic of CZS, the emphasis should remain on understanding how viral breaching of placental barriers creates fetal vulnerability rather than detailing the downstream neurological pathogenesis that occurs once the virus reaches fetal tissues. The mechanisms underlying Zika virus-induced microcephaly are complex and likely involve both direct viral effects on neural progenitor cells and indirect effects mediated through placental dysfunction (198). Understanding these mechanisms has implications for other viral infections that may cause similar neurodevelopmental abnormalities.

### 5.3 Cytomegalovirus

CMV is the most common cause of congenital viral infection in developed countries. It helps us understand chronic viral infections in the placenta. The virus can infect placental cells and cause long-lasting infections during pregnancy (23, 199). CMV infection results in specific placental issues, such as villous immaturity, chronic villitis, and hemosiderin buildup (**Figure 3**). The virus can also harm placental blood vessels, leading to blockages and tissue death, which affects the delivery of oxygen and nutrients to the fetus (23). The factors influencing the severity of congenital CMV infection are not fully understood. They likely include when the mother gets infected, the characteristics of the viral strain, and the mother’s immune health. Primary infections in mothers usually carry a higher risk of transmission compared to reactivating latent infections. However, severe congenital disease can happen in both cases (23).

CMV exemplifies sophisticated placental invasion strategies through its multifaceted tropism for diverse placental cell populations, initially targeting maternal decidual cells and macrophages before progressing to infect cytotrophoblasts, syncytiotrophoblasts, and placental mesenchymal cells including Hofbauer cells (placental macrophages) and endothelial cells within fetal capillaries. CMV employs receptor-mediated endocytosis through multiple cellular entry points, including platelet-derived growth factor receptor-α (PDGFR-α), epidermal growth factor receptor (EGFR), and integrins, enabling efficient cellular invasion across different placental compartments (88). The virus demonstrates remarkable dissemination capabilities through both cell-to-cell spread via intercellular bridges and hematogenous dissemination through infected maternal leukocytes that traverse placental vasculature, while simultaneously establishing persistent infections in placental stromal cells that serve as viral reservoirs throughout gestation (200). Vertical transmission mechanisms involve direct viral passage across compromised syncytiotrophoblast barriers, infection of villous capillary endothelium leading to fetal blood contamination, and ascension through amniotic fluid following placental viral shedding, with transmission efficiency varying dramatically based on gestational timing (201). Other viral pathogens including HSV, varicella-zoster virus (VZV), and parvovirus B19 employ analogous but distinct mechanisms: HSV primarily targets decidual cells and ascends through cervical infection routes, VZV demonstrates strong tropism for placental endothelial cells causing characteristic hemorrhagic villitis, while parvovirus B19 specifically targets erythroid precursor cells within placental and fetal hematopoietic tissues, leading to severe fetal anemia and hydrops fetalis through disruption of erythropoiesis rather than direct placental structural damage (200).

### 5.4 Other viral pathogens

Numerous other viral pathogens can infect placental tissues and cause maternal-fetal transmission, each with unique mechanisms and clinical consequences. Parvovirus B19 causes severe fetal anemia through infection of erythroid precursors and can lead to hydrops fetalis and fetal death (202). Varicella-zoster virus can cause congenital varicella syndrome, characterized by distinctive skin, limb, and neurological abnormalities (203).

HBV represents an important cause of perinatal transmission, particularly during delivery when fetal exposure to maternal blood is highest (204). HIV can cross the placental barrier, but transmission rates have been dramatically reduced through antiretroviral therapy and other interventions (27).

This schematic compares how major viral pathogens exploit placental pathways, activate immune signaling, and drive pregnancy complications. Viral entry and receptor usage:SARS-CoV-2: Uses ACE2/TMPRSS2 on syncytiotrophoblasts; Zika virus (ZIKV): Engages AXL/TIM receptors on trophoblasts and macrophages; HIV: Enters via CD4/CCR5 or syncytin-mediated fusion; CMV: Utilizes PDGFRα and integrins to infect CTBs. PRRs (TLRs, RIG-I, NOD2) trigger NF-κB and IRFs, inducing IFNs, TNF-α, and IL-6, and activating the NLRP3 inflammasome. This drives pyroptosis (via caspase-1) and release of IL-1β/IL-18, amplifying placental inflammation. The cumulative effect of viral entry, innate/adaptive immune dysregulation, and cellular stress responses (oxidative stress, ER stress, pyroptosis) manifests as: Placental damage – villous inflammation, syncytial injury, trophoblast apoptosis; Maternal complications – hemorrhage, systemic cytokine storm, immune exhaustion; Fetal outcomes – growth restriction, congenital malformations (neurological, cardiac), stillbirth, preterm delivery, and impaired neurodevelopment.

## 6 Clinical consequences and pregnancy outcomes from virus infection

### 6.1 Acute pregnancy complications

**Figure 4:**
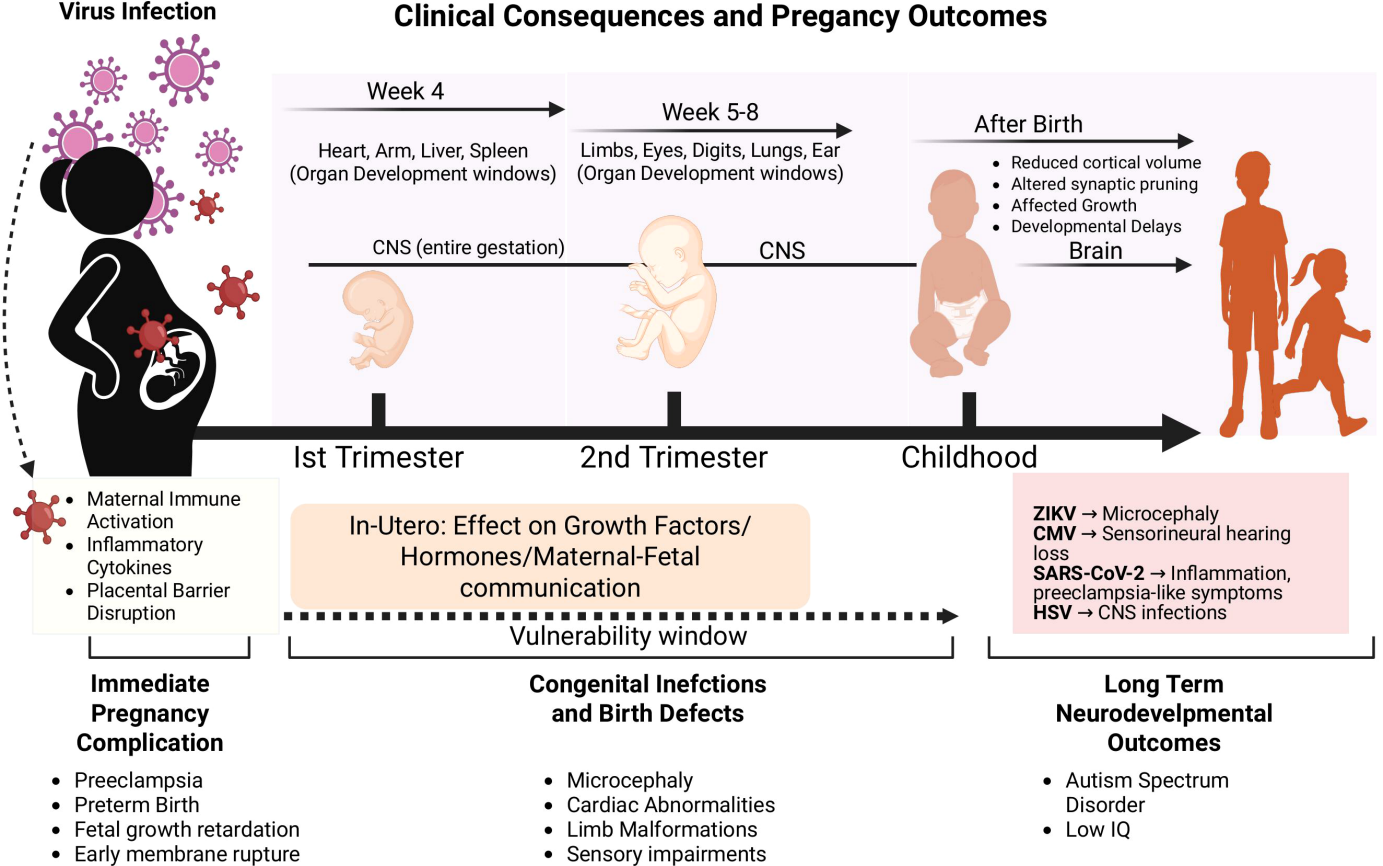
Clinical consequences and pregnancy outcomes.

Infections of the placenta caused by viruses can lead to several immediate issues during pregnancy that threaten the health of both the mother and the fetus (**Figure 4**). Inflammation and dysfunction of the placenta may result in conditions similar to preeclampsia, characterized by high blood pressure, protein in the urine, and widespread maternal inflammation (27). These problems can arise from the direct effects of viruses on the placenta’s blood vessels or from systemic reactions triggered by cytokines. Another common result of viral placental infections is fetal growth restriction (FGR), which occurs due to reduced placental transport capabilities and lower delivery of oxygen and nutrients to the fetus (205). The severity of FGR depends on several factors, including how severe the placental infection is, the gestational age when the disease occurs, and how long the infection lasts. Inflammation caused by viruses can lead to preterm labor and delivery because of the production of prostaglandins and other substances that encourage uterine contractions (24). Moreover, some viruses may directly infect fetal membranes, resulting in the premature rupture of membranes.

### 6.2 Long-term neurodevelopmental outcomes

The long-term effects of congenital viral infections on brain development are an important area of research. This focus has grown due to the Zika virus outbreak and concerns about how COVID-19 may affect fetal brain growth. Even when no clear structural issues are visible at birth, subtle differences in development can show up as children grow and their cognitive skills are assessed (27, 142). Autism spectrum disorders, attention deficit hyperactivity disorder, and learning disabilities are linked to various congenital viral infections (**Figure 4**) (206–208). However, proving direct cause-and-effect relationships is difficult due to the complex nature of these conditions. Long-term studies that track children exposed to viral infections before birth are essential for understanding these lasting effects.

This schematic timeline illustrates the potential adverse outcomes of viral infections during pregnancy, highlighting immediate, congenital, and long-term neurodevelopmental effects. The left panel shows maternal infection leading to immediate pregnancy complications such as preeclampsia, preterm birth, fetal growth restriction, and premature rupture of membranes due to placental inflammation and dysfunction. The central panel indicates that congenital infections and birth defects vary with gestational timing, including microcephaly, cardiac abnormalities, limb malformations, and sensory impairments, particularly when infection occurs during the first or early second trimester when organogenesis and critical developmental processes are underway. The right panel depicts long-term neurodevelopmental outcomes, including autism spectrum disorder and reduced cognitive function, which may manifest during childhood even in the absence of overt structural abnormalities at birth. This figure integrates mechanistic insights into viral-induced placental pathology, fetal vulnerability by gestational stage, and epidemiological findings on developmental outcomes, emphasizing the importance of preventing and managing maternal viral infections to safeguard both immediate and lifelong health of the offspring.

### 6.3 Congenital viral infections and spectrum of birth defects

Congenital viral infections are a significant cause of fetal morbidity and mortality, leading to a wide range of structural deformities, functional limitations, and long-lasting developmental challenges that differ substantially based on the specific pathogen involved, the timing of infection during gestation, and the unique susceptibility profiles of each fetus. The clinical presentations affect various organ systems, with neurological disorders representing the most severe outcomes due to the heightened vulnerability of the developing central nervous system to viral damage (160). The period during which a viral infection occurs during pregnancy is crucial in determining both the severity and types of congenital defects that may arise, with infections in the first trimester, particularly weeks 3 to 12 when organ formation takes place, resulting in the most serious and varied spectrum of malformations. Defects in the neural tube, such as spina bifida, anencephaly, and encephalocele, occur due to viral disruptions of the processes responsible for neural tube closure in weeks 3 and 4 of gestation (209). Microcephaly, one of the most identifiable outcomes of congenital viral infections, arises through several mechanisms, including direct viral damage to neural progenitor cells, interference with neuronal migration, and disruption of cortical development. Additional brain abnormalities frequently linked to congenital viral infections include lissencephaly (a smooth brain), polymicrogyria (excessive folding of the cortex), cerebellar hypoplasia, and ventriculomegaly, each signifying specific disturbances in neurodevelopmental processes during key morphogenetic stages (210).

## 7 Diagnostic approaches and challenges

### 7.1 Maternal diagnostic testing

Accurate diagnosis of viral infections during pregnancy is crucial for effective clinical management and informed counseling regarding transmission risks and potential pregnancy outcomes. However, standard diagnostic approaches may not always provide sufficient information about placental infection status or transmission risk. Serological testing can identify maternal infection and provide information about the timing (primary vs. recurrent infection), but may not accurately predict placental involvement or risk of fetal transmission. Molecular testing of maternal blood or other specimens can detect active viral replication, but it may miss cases where the viral infection is limited to placental tissues (113).

Maternal diagnostic testing for viral infections during pregnancy requires a multifaceted approach that recognizes the inherent limitations of traditional serological methods, particularly for viruses with brief viremic phases or those that rapidly establish latency, necessitating integration of molecular, immunological, and cutting-edge diagnostic platforms. While serological testing remains foundational through detection of virus-specific IgM antibodies indicating acute infection and IgG avidity assays distinguishing recent from remote infections, these approaches prove inadequate for pathogens like CMV and Epstein-Barr virus (EBV) that exhibit transient viremia lasting only days to weeks, requiring real-time PCR detection of viral DNA in maternal blood, urine, or saliva during narrow diagnostic windows (211). Advanced molecular diagnostics now encompass next-generation sequencing (NGS) for comprehensive pathogen detection, digital droplet PCR (ddPCR) for ultra-sensitive viral quantification, and multiplex PCR panels enabling simultaneous screening for multiple viral pathogens with enhanced sensitivity and specificity (212). Revolutionary diagnostic technologies include analysis of cell-free circulating DNA (cfDNA) that can detect both maternal and fetal viral sequences, mitochondrial DNA (mtDNA) analysis as biomarkers of cellular stress and viral-induced tissue damage, and exosome-based diagnostics that capture virus-derived cargo including viral proteins, nucleic acids, and microRNAs released from infected placental cells, providing real-time insights into placental viral activity and fetal risk assessment (213). Emerging platforms such as CRISPR-based diagnostics (SHERLOCK, DETECTR) offer point-of-care viral detection with laboratory-grade accuracy (214, 215). At the same time, proteomics and metabolomics approaches identify viral infection signatures through host response patterns. Artificial intelligence-enhanced diagnostic algorithms combine various biomarker streams to assess the risk of vertical transmission and refine clinical decision-making, collectively evolving maternal viral diagnostics from reactive serological testing to proactive, precision-based monitoring that can identify subclinical infections and forecast fetal outcomes with remarkable accuracy.

The development of biomarkers that specifically reflect placental infection or damage represents a critical research priority. Placental proteins, nucleic acids, or other markers released into maternal circulation could potentially provide real-time information about placental health and viral infection status.

### 7.2 Fetal diagnostic testing for viral infection

Prenatal diagnosis of fetal viral infections typically relies on amniocentesis to detect viral nucleic acids in amniotic fluid (216). However, the sensitivity and specificity of these tests vary depending on the virus, gestational age at the time of testing, and the timing relative to maternal infection. Advanced fetal imaging techniques, including detailed ultrasound and magnetic resonance imaging, can detect structural abnormalities associated with congenital infections but may miss more subtle effects on fetal development (217, 218). The integration of multiple diagnostic modalities is often necessary for comprehensive fetal assessment.

Amniocentesis presents several critical limitations in the context of maternal viral infections, most notably its inherently invasive nature that carries procedure-related risks, including pregnancy loss, bleeding, infection, and premature rupture of membranes, while paradoxically creating potential pathways for iatrogenic vertical transmission of viruses that may not have naturally breached the placental barrier (219, 220). The procedural technique itself can inadvertently facilitate viral transmission through needle tract contamination, where the amniocentesis needle traverses maternal tissues harboring virus (such as infected decidua or maternal blood) before entering the amniotic cavity, effectively bypassing natural placental protective mechanisms and introducing pathogens directly into the fetal environment (221). Additionally, timing constraints significantly limit diagnostic utility, as amniocentesis cannot be safely performed before 15–18 weeks’ gestation, often occurring weeks after critical periods of organogenesis when teratogenic damage may have already occurred, while early performance increases risks of false-negative results since sufficient time may not have elapsed for viral replication and shedding into amniotic fluid. Furthermore, the procedure’s diagnostic accuracy is compromised by sampling limitations, as amniotic fluid viral detection depends on active fetal viral shedding through urine or respiratory secretions, meaning negative results do not definitively exclude fetal infection, particularly for viruses with intermittent shedding patterns or those that establish latent infections in fetal tissues without significant amniotic fluid contamination, necessitating careful risk-benefit analysis and consideration of alternative diagnostic approaches.

### 7.3 Placental examination and pathology for diagnosis

A detailed examination of placental tissues following delivery can provide valuable information about viral infections and their impact on placental function. However, standard histopathological examination may miss focal infections or chronic changes that do not produce obvious inflammatory responses (113).

Placental pathological examination employs a comprehensive multi-modal approach combining gross morphological assessment, histopathological analysis, immunohistochemistry, and molecular techniques to identify virus-induced alterations that provide crucial diagnostic and prognostic information. Pathologists systematically evaluate gross placental features including weight, dimensions, surface lesions, and villous architecture before performing detailed microscopic examination targeting specific viral-associated changes: chronic villitis characterized by lymphocytic infiltration within chorionic villi (particularly prominent in CMV and HSV infections), acute chorioamnionitis with neutrophilic invasion of fetal membranes, necrotizing funisitis affecting umbilical cord vessels, and distinctive inclusion bodies such as CMV’s characteristic “owl’s eye” intranuclear inclusions and enlarged cells with amphophilic cytoplasm (113). Advanced analyses include identification of pathognomonic features like dystrophic calcifications in CMV placentitis, fibrin deposits indicating villous necrosis and thrombotic vasculopathy, hemosiderin-laden macrophages suggesting chronic bleeding, and avascular villi reflecting fetal vascular compromise (222). Immunohistochemical staining with virus-specific antibodies enables direct viral antigen detection within infected cells, while *in-situ* hybridization localizes viral nucleic acids to specific cellular compartments, and electron microscopy can identify viral particles and ultrastructural cellular damage (223, 224). This comprehensive pathological assessment provides invaluable clinical information including confirmation of intrauterine viral infection when maternal and fetal serological results are ambiguous, estimation of infection timing and duration based on inflammatory patterns and tissue remodeling, prediction of fetal outcomes through correlation of placental lesion severity with neurodevelopmental prognosis, guidance for neonatal management and monitoring protocols, and establishment of recurrence risk for future pregnancies, making placental pathology an indispensable component of perinatal viral infection evaluation that bridges maternal infection status with fetal impact assessment.

Advanced techniques, including immunohistochemistry, *in situ* hybridization, and molecular testing of placental tissues, can enhance the detection of viral infections and provide insights into the mechanisms of placental pathology (113). Single-cell sequencing and other genomic approaches are beginning to reveal the heterogeneous effects of viral infections on different placental cell populations (10, 215).

## 8 Therapeutic interventions and prevention strategies

### 8.1 Antiviral therapies

The development of safe and effective antiviral therapies for use during pregnancy represents a major clinical need. Still, it poses significant challenges due to concerns about fetal safety and the unique pharmacokinetic properties of pregnancy. However, pregnancy introduces unique challenges for antiviral pharmacotherapy due to altered pharmacokinetics, hemodynamic changes, and concerns about teratogenicity and fetal drug exposure (225).

Antiviral therapies include pregnancy-safe agents such as acyclovir and valacyclovir for herpes simplex virus infections, ganciclovir and valganciclovir for severe CMV disease (though with limited placental penetration and potential teratogenic concerns), and oseltamivir for influenza, while newer agents like letermovir for CMV and direct-acting antivirals for hepatitis C show promise but require extensive safety evaluation in pregnancy (225). Emerging placenta-specific drug delivery systems, including nanoparticle formulations, ligand-targeted liposomes, and exosome-based carriers, are under investigation to enhance antiviral activity locally while minimizing systemic fetal exposure. Monoclonal antibodies represent another promising avenue, particularly for viruses where neutralization of circulating virions or blockade of placental entry receptors is feasible. Owing to their large molecular size and FcRn-mediated transport regulation, antibodies generally exhibit limited and developmentally regulated transplacental transfer, which may provide maternal protection while conferring passive neonatal immunity without significant teratogenic risk (226). Future strategies integrating long-acting antiviral biologics with precision delivery platforms could substantially improve therapeutic outcomes in this vulnerable population.

### 8.2 Immunomodulatory approaches

Given the important role of immune responses in antiviral protection and placental issues, immunomodulatory therapies offer a hopeful option for treating viral infections during pregnancy. Interferon therapies have been used for certain viral infections. Immunomodulatory approaches encompass hyperimmune globulin preparations (such as CMV-IVIG) that provide passive antibody protection and may reduce vertical transmission rates, interferon therapy for specific indications despite pregnancy category concerns, and emerging monoclonal antibody therapies targeting specific viral epitopes with enhanced safety profiles. Type I interferons and related cytokine-based therapies demonstrate potent antiviral effects *in vitro*; however, systemic administration is constrained by concerns regarding excessive inflammation, impaired placental perfusion, and potential developmental toxicity (166). Novel approaches are focusing on spatially and temporally restricted modulation of innate signaling pathways, such as selectively enhancing trophoblast-specific interferon responses or employing agents that bias the balance toward antiviral immunity while minimizing tissue-damaging inflammation. For example, targeted modulation of adaptor proteins downstream of TLRs or RIG-I–like receptors in syncytiotrophoblasts could preserve antiviral defenses while limiting bystander inflammation in the villous core (227). Similarly, small-molecule inhibitors or biologics that block detrimental cytokines (e.g., TNF-α, IL-6) without suppressing protective interferon responses are being explored as precision therapies (228). Collectively, such interventions reflect a paradigm shift toward fine-tuned immunomodulation at the maternal–fetal interface rather than broad immunosuppression.

### 8.3 Vaccination strategies

Vaccination represents the most powerful and cost-effective means of preventing viral infections during pregnancy, but its implementation faces significant scientific and clinical challenges. Pregnancy-associated immunological adaptations—including skewing toward regulatory T-cell responses, altered B-cell dynamics, and changes in Fc receptor-mediated antibody transfer—can modify vaccine efficacy and durability (77). Maternal immunization not only protects the mother but also enables passive antibody transfer across the placenta, conferring neonatal protection in the early months of life. However, optimization of vaccination schedules remains essential, as antibody titers and transfer efficiency vary with gestational age (229). The contraindication of live-attenuated vaccines during pregnancy due to risks of vertical transmission and teratogenicity further restricts available options, underscoring the need for inactivated, subunit, or nucleic acid-based platforms with robust safety data. Recent advances in adjuvant design highlight the possibility of tailoring immune activation to the pregnancy context—for instance, using adjuvants that favor humoral responses without excessive Th1 activation or designing vaccines that induce mucosal immunity to intercept pathogens at their portals of entry (229). The development of pregnancy-specific vaccination strategies, alongside rigorous safety monitoring and clinical trial inclusion of pregnant populations, thus represents a critical frontier in maternal–fetal medicine.

## 9 Emerging technologies and research approaches

### 9.1 Advanced placental models

Traditional approaches to studying placental viral infections have relied heavily on animal models and ex vivo human placental tissues, both of which have significant limitations for understanding human placental virology. Recent advances in tissue engineering and stem cell biology have enabled the development of more sophisticated *in vitro* models that better recapitulate human organ structure and function, including placenta (230–235). Placental organoids derived from human trophoblast stem cells or induced pluripotent stem cells can model key aspects of placental development and function, allowing for controlled studies of viral infections. These three-dimensional culture systems maintain many of the cellular interactions and tissue architecture present in native placental tissues and can be used to study viral tropism, pathogenesis, and therapeutic interventions (37, 230, 236, 237). These three-dimensional models preserve cellular heterogeneity and paracrine signaling networks absent in two-dimensional systems, making them particularly valuable for dissecting virus–host interactions and testing therapeutic interventions. Complementarily, placenta-on-a-chip devices represent another innovative approach that combines microfluidics with human placental cells to create dynamic models of maternal-fetal transport and barrier function (231). These systems can model the effects of viral infections on placental transport and barrier integrity in real-time, making them useful for testing therapeutic interventions.

### 9.2 Single-cell and spatial omics-based technology

High-resolution omics approaches are revolutionizing our understanding of cellular heterogeneity in the placenta and its response to viral infection. Single-cell RNA sequencing has revealed distinct trophoblast, immune, and stromal populations, including rare subsets potentially acting as viral reservoirs or amplifiers of inflammation (72). Integrative multi-omics approaches, including single-cell proteomics and chromatin accessibility assays, are uncovering transcriptional and epigenetic rewiring of infected cells, offering mechanistic insights into viral persistence and host defense. They also reveal the molecular processes behind viral infections. Spatially resolved transcriptomics and proteomics add a critical dimension by mapping infection sites and immune responses within placental microenvironments, thereby linking cellular localization with pathophysiological outcomes such as villitis, syncytial damage, and impaired nutrient transfer (205, 238–241). This provides insights into how diseases spread and how tissue structure influences viral infections. Together, these approaches bridge molecular signatures with histopathological manifestations, advancing precision mapping of viral pathogenesis.

### 9.3 Advanced imaging and computational approaches

Next-generation imaging modalities are providing unprecedented resolution of viral–placental interactions at both structural and dynamic levels. Super-resolution microscopy and cryo-electron microscopy have elucidated mechanisms of viral entry, endosomal trafficking, and subcellular replication niches, while correlative light and electron microscopy (CLEM) enables real-time visualization of infection dynamics within intact tissue (37, 242).

Multiplexed imaging approaches further capture the spatial organization of infected and bystander cells, linking infection to local immune crosstalk and barrier integrity. In parallel, computational modeling is increasingly employed to integrate multi-omic, imaging, and functional datasets, producing predictive frameworks for vertical transmission risks and therapeutic efficacy. Such in silico models provide systems-level perspectives on viral kinetics, immune evasion, and barrier disruption, while highlighting experimental gaps that require targeted investigation (90). Collectively, these converging technologies represent a paradigm shift in placental virology, moving from descriptive observations to predictive, mechanistic frameworks.

## 10 Future directions and research priorities

### 10.1 Understanding viral transmission determinants

Despite decades of research, the factors that determine whether maternal viral infections will result in placental infection and fetal transmission remain incompletely understood. Future research should focus on identifying the viral, maternal, and placental factors that influence transmission risk, as well as developing predictive models to guide clinical management. Large-scale studies that combine detailed viral characterization, maternal immune profiling, and placental analysis are needed to identify biomarkers of transmission risk and develop personalized approaches to managing viral infections during pregnancy. These studies should also investigate the role of co-infections, maternal comorbidities, and environmental factors in modulating transmission risk.

### 10.2 Developing placenta-specific therapeutics

The unique anatomical and physiological characteristics of the placenta require specialized therapeutic approaches that differ from those used in other tissues. Future research should focus on developing drug delivery systems that can specifically target placental tissues while minimizing fetal exposure. Nanoparticle-based delivery systems, placenta-targeting ligands, and other innovative approaches have the potential to enhance the therapeutic index of antiviral agents and other interventions. The development of placenta-specific therapeutics will require close collaboration between virologists, pharmacologists, and maternal-fetal medicine specialists.

### 10.3 Addressing global health disparities

Viral infections during pregnancy disproportionately affect women in low- and middle-income countries, where access to prevention, diagnosis, and treatment may be limited. Future research should prioritize the development of low-cost, point-of-care diagnostic tests and interventions that can be implemented in resource-limited settings. Global surveillance systems for viral infections during pregnancy are needed to identify emerging threats and monitor the effectiveness of prevention and treatment strategies. These systems should integrate data from multiple countries and regions to provide comprehensive assessments of viral threats to maternal and fetal health.

## 11 Conclusion

The placenta is increasingly recognized as a dynamic immunological and metabolic hub rather than a passive barrier, yet viral transmission across this interface remains poorly understood. Insights gained during the COVID-19 pandemic underscore the urgency of delineating the mechanisms that regulate placental susceptibility, viral entry, and maternal–fetal transmission. Moving forward, research should prioritize (i) defining receptor–ligand interactions and signaling pathways that govern viral tropism, (ii) developing sensitive diagnostics and safe antiviral strategies tailored to pregnancy, and (iii) translating emerging experimental models—placental organoids, placenta-on-a-chip, and single-cell spatial omics—into clinically actionable tools. Equally important is the integration of computational modeling and advanced imaging to bridge molecular discoveries with *in vivo* pathophysiology. Achieving these objectives will require sustained collaboration between basic scientists, clinicians, and public health experts. Ultimately, the goal of placental virology research is to generate predictive risk assessment tools and targeted interventions that safeguard maternal–fetal health while illuminating fundamental principles of human development and lifelong disease susceptibility. As we continue to interrogate the “black box” of maternal–fetal biology, discoveries in placental virology promise not only to reduce the burden of viral infections in pregnancy but also to expand our understanding of placental function and its enduring influence on health across the lifespan.
